# Rare epigenetic alterations are conserved across hematopoietic differentiation stages after mycobacterial infection

**DOI:** 10.1172/jci.insight.193686

**Published:** 2025-12-09

**Authors:** Brandon T. Tran, Pamela N. Luna, Ruoqiong Cao, Duy T. Le, Apoorva Thatavarty, Laure Maneix, Bailee N. Kain, Scott Koh, Andre Catic, Katherine Y. King

**Affiliations:** 1 Graduate Program in Cancer and Cell Biology,; 2 Department of Pediatrics, Division of Infectious Disease, Texas Children’s Hospital and Baylor College of Medicine,; 3 Stem Cells and Regenerative Medicine Center,; 4 Department of Molecular and Human Genetics,; 5 Graduate Program in Immunology and Microbiology,; 6 Graduate Program in Molecular and Human Genetics,; 7 Huffington Center of Aging, and; 8 Dan L. Duncan Comprehensive Cancer Center, Baylor College of Medicine, Houston, Texas, USA.; 9 Division of Experimental Hematology, Department of Pediatrics, Cincinnati Children’s Hospital Medical Center, Cincinnati, Ohio, USA.; 10 Department of Molecular and Cellular Biology, Baylor College of Medicine, Houston, Texas, USA.

**Keywords:** Hematology, Immunology, Epigenetics, Innate immunity, Memory

## Abstract

Infection leads to durable cell-autonomous changes in hematopoietic stem and progenitor cells (HSPCs), resulting in production of innate immune cells with heightened immunity. The mechanisms underlying this phenomenon, termed central trained immunity, remain poorly understood. We hypothesized that infection induces histone modifications leading to changes in chromatin accessibility that are conserved during differentiation from HSPCs to myeloid progenitors and monocytes. We conducted genome-wide surveillance of histone marks H3K27ac and H3K4me3 and chromatin accessibility in hematopoietic stem cells, multipotent progenitor 3, granulocyte-monocyte progenitors, and monocytes and macrophages of naive and *Mycobacterium avium*–infected mice. IFN signaling pathways and related transcription factor binding motifs including IRFs, NF-κB, and CEBP showed increased activating histone marks and chromatin accessibility across cell types. However, histone marks and increased chromatin accessibility were conserved at only a few loci, notably *Irf1* and *Gbp6*. Knock out of *IRF1* disrupted enhanced mitochondrial respiration and bacterial killing in human monocyte cell lines, while *GBP6-*KO monocyte cell lines showed dysregulated mitochondrial respiration. In summary, this study identifies IRF1 and GBP6 as 2 key loci at which infection-induced systemic inflammation leads to epigenetic changes that are conserved from HSPCs to downstream monocytes, providing a mechanistic avenue for central trained immunity.

## Abstract 2

Innate immune cells like macrophages harbor long-term memory by unknown mechanisms. We identify epigenetic changes in hematopoietic stem and progenitor cells retained in downstream macrophages.

## Graphical abstract

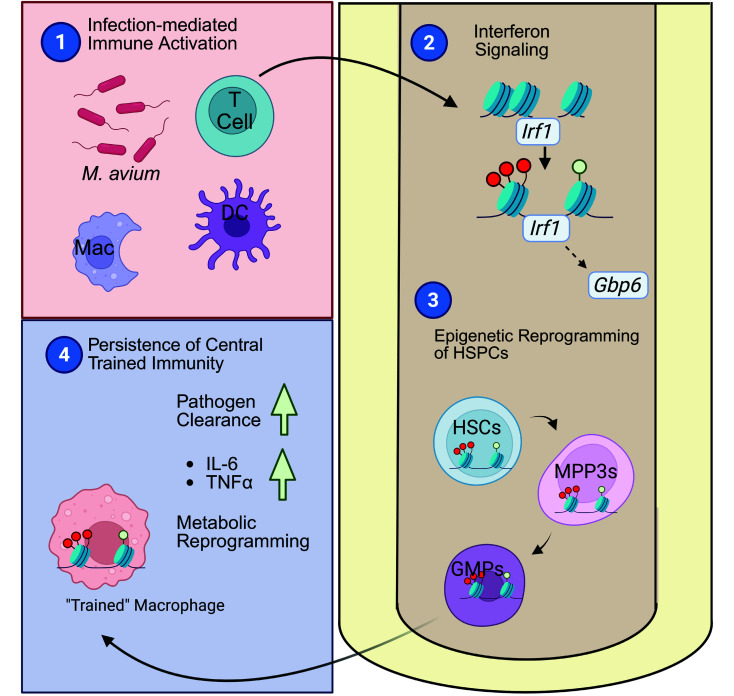

## Introduction

The adaptive and innate immune systems work in concert to prevent and fight infections. While the adaptive immune system traditionally has been considered the seat of immunological memory through the generation of memory lymphoid cells, emerging evidence indicates that innate immune cells, such as macrophages and neutrophils, contribute to immune memory (1, 2). Indeed, innate immune cells, when challenged by pathogens or pathogen-associated molecular patterns (PAMPs), undergo epigenetic reprogramming to elicit heightened secondary immune responses (1–3). Hallmarks of the innate immune memory immunophenotype include enhanced oxidative phosphorylation and lipid metabolism, proinflammatory cytokine production, phagocytosis, and pathogen clearance (4–7). Epidemiological studies and experimental vaccination trials in humans have further demonstrated that vaccination can confer improved nonspecific immune responses by innate effector cells (8–10).

Innate memory immune responses have been recorded for as long as 1 year after the initiating event in human studies, beyond the lifetime of most circulating innate immune cells (9, 11). This observation led to the speculation that long lived hematopoietic stem and progenitor cells (HSPCs) play a crucial role in the development and maintenance of innate immune memory. HSPCs, which consist of hematopoietic stem cells (HSCs) and 4 types of multipotent progenitors (MPPs 1–4), are responsible for lifelong blood production. HSPCs respond to environmental cues such as inflammatory cytokines, infection, diet, and aging (12–16). Furthermore, HSCs have been shown to harbor sustained epigenetic changes after infection (17–20). Several studies using functional assays and next-generation sequencing techniques found that HSPCs can be epigenetically altered to produce downstream immune cells with enhanced metabolism, cytokine production, and bacterial clearance (18, 21, 22). Even after serial transplantation into immunologically naive recipient mice, HSPCs continued to produce downstream macrophages with enhanced cross-protective functional capacity (18, 23, 24). Thus, inflammation-responsive HSPCs may be reprogrammed to promote increased myeloid differentiation and produce trained innate immune cells, a concept termed central trained immunity (CTI). Fully elucidating the specific mechanisms by which HSPCs confer CTI has, therefore, become a critical avenue by which to improve host immunity.

CTI is thought to be generated by inflammation-induced epigenetic reprogramming of histone modifications and other chromatin-modifying programs that poise the chromatin for faster and stronger immune responses. Studies have found that HSPCs and innate immune cells acquire activating histone modifications — including H3K4 mono- and trimethylation (H3K4me1, H3K4me3), and H3K27 acetylation (H3K27ac) — to promote myeloid differentiation and innate immune responses (18, 19, 21). However, how changes at the stem and progenitor level lead to changes in downstream cells remains unclear (3, 18, 25, 26). One potential mechanism is direct conservation of epigenetic modifications acquired by HSPCs (1, 2). Indeed, histone modifications can be passed down during DNA replication and are critical in cell function (27–30). Therefore, histone modifications may play a role in CTI generation, but few studies have provided direct evidence supporting this.

Here, we assessed whether sites of H3K4me3 and H3K27ac, modifications frequently enriched in CTI and reported to be conserved through cell division and DNA replication (29), and chromatin accessibility are conserved between HSPCs and innate immune cells after inflammatory stress. Utilizing a *Mycobacterium avium* infection model of CTI, we found that increased IFN-β cytokine signaling and associated transcription motifs were conserved across all cell types, underscoring the central role of cytokine signaling as a mediator of CTI. Activating histone modifications and chromatin accessibility changes were only conserved at a small number of genetic loci, suggesting that these loci are relevant in the establishment of CTI. 

## Results

### M. avium infection induces long lasting central HSPC trained immunity to promote altered macrophage immune and metabolic function.

**Figure 1:**
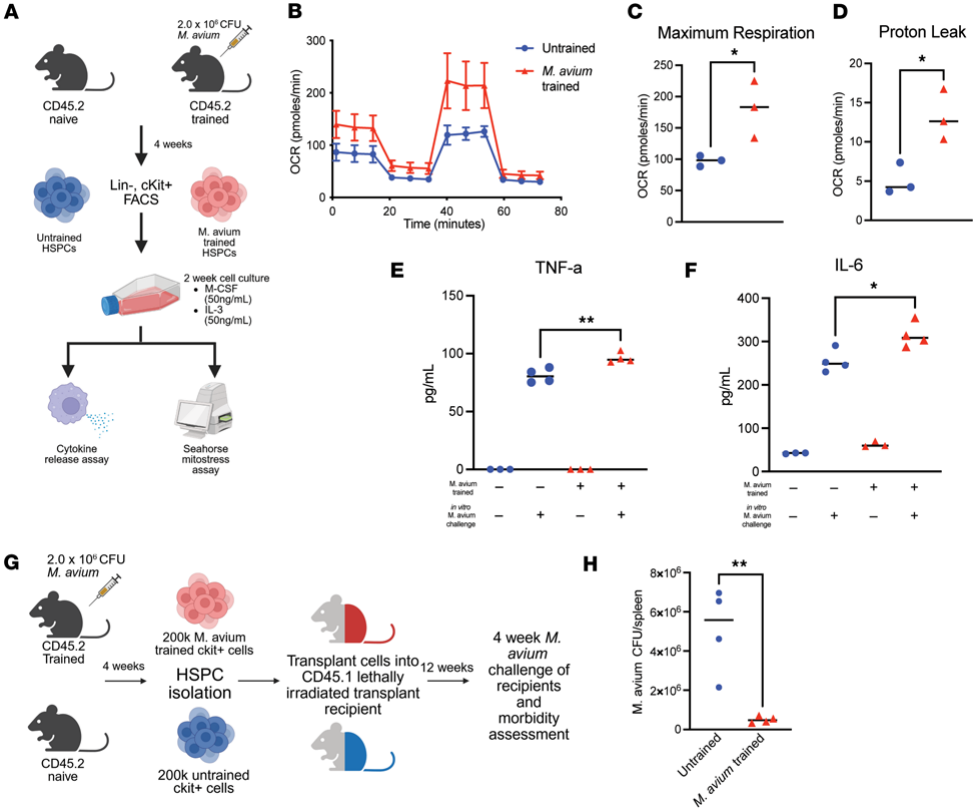
*Mycobacterium avium* infection promotes HPSC central trained immunity with enhanced macrophage immune and metabolic function. (**A**) Experimental design for generating bone marrow–derived macrophages (BMDMs) from hematopoietic stem and progenitor cells (HSPCs). Cells were sorted from pooled mouse bone marrow samples (*n* = 5–7 per group), cKit-enriched, and sorted for lineage^–^ cells. BMDMs generated from sorted cells were counted and seeded for respective assays after culture. (**B**) Seahorse Mitostress test assay in *M*. *avium–*trained BMDMs (red) and naive/untrained BMDMs (blue). Experiment was repeated twice. (**C** and **D**) Calculated maximum oxygen consumption rate (**C**) and proton leak (**D**) of *M*. *avium–*trained and naive BMDMs. (**E** and **F**) TNF-α (**E**) and IL-6 (**F**) secreted by *M*. *avium–*trained and naive BMDMs. Cytokine release assay performed once. (**G**) Experimental schematic for trained immunity chimeric transplant and challenge model. In total, 200,000 CD45.2 cKit^+^ cells from naive or *M*. *avium–*trained mice were transplanted with 200,000 CD45.1 rescue marrow into lethally irradiated CD45.1 recipients, which were challenged with *M*. *avium* 12 weeks after transplant. (**H**) Splenic *M*. *avium* colony forming units counts from *M*. *avium–*trained and naive challenged transplant recipients. *n* = 4–6 per experiment, representative findings from 2 independent experiments. Mean ± SEM shown per experiment. For statistical analysis (**C**, **D**, and **H**), 2-tailed Student’s unpaired *t* test. **E** and **F** used ordinary 1-way ANOVA. *P < 0.05, **P < 0.01.

In prior work, we found that *M*. *avium–*stimulated HSPCs generate bone marrow–derived macrophages (BMDMs) with heightened immunophenotypes, including increased cytokine production, bacterial clearance, and metabolic rewiring (22) (Figure 1A). Here, we confirmed those findings by showing that BMDMs derived from *M*. *avium–*trained HSPCs have increased maximal oxygen consumption rate (OCR) (*P* = 0.0355) and proton leak (*P* = 0.0207) (Figure 1, B–D). Increased OCR is linked to higher utilization of oxidative phosphorylation, while increased mitochondrial proton leak is associated with damaged mitochondria and ROS production (31, 32). We also found that *M*. *avium–*trained BMDMs produced significantly increased TNF-α (*P* = 0.002) and IL-6 (*P* = 0.0112), proinflammatory cytokines that can promote HSPC differentiation (33, 34) and commonly known to be secreted by trained macrophages (6, 24) (Figure 1, E and F).

To better understand how *M*. *avium* training affects host protection in vivo, we performed competitive transplants of HSPCs that were untrained or trained with *M*. *avium* infection into naive recipients (Figure 1G). Both groups produced trilineage hematopoiesis 3 months later, indicating engraftment in the transplant recipients (Supplemental Figure 1, A–C; supplemental material available online with this article; https://doi.org/10.1172/jci.insight.193686DS1). Recipients were then challenged with *M*. *avium* for 4 weeks, and splenic bacterial burden was measured as an indicator of host protection. While we did not detect any difference in mortality between the groups, we found significantly lower bacterial burden within spleens of *M*. *avium–*trained HSPC recipients compared with untrained controls (*P* = 0.006) (Figure 1H), validating prior findings (8, 11, 22, 35). Collectively, our work suggests that infection and inflammation reprogram HSPCs to produce trained innate immune cells with improved host protection capacity (9, 11, 21, 23, 24).

### M. avium–infected HSPCs and monocytes/macrophages exhibit restricted overlap of H3K27ac acquisition.

**Figure 2:**
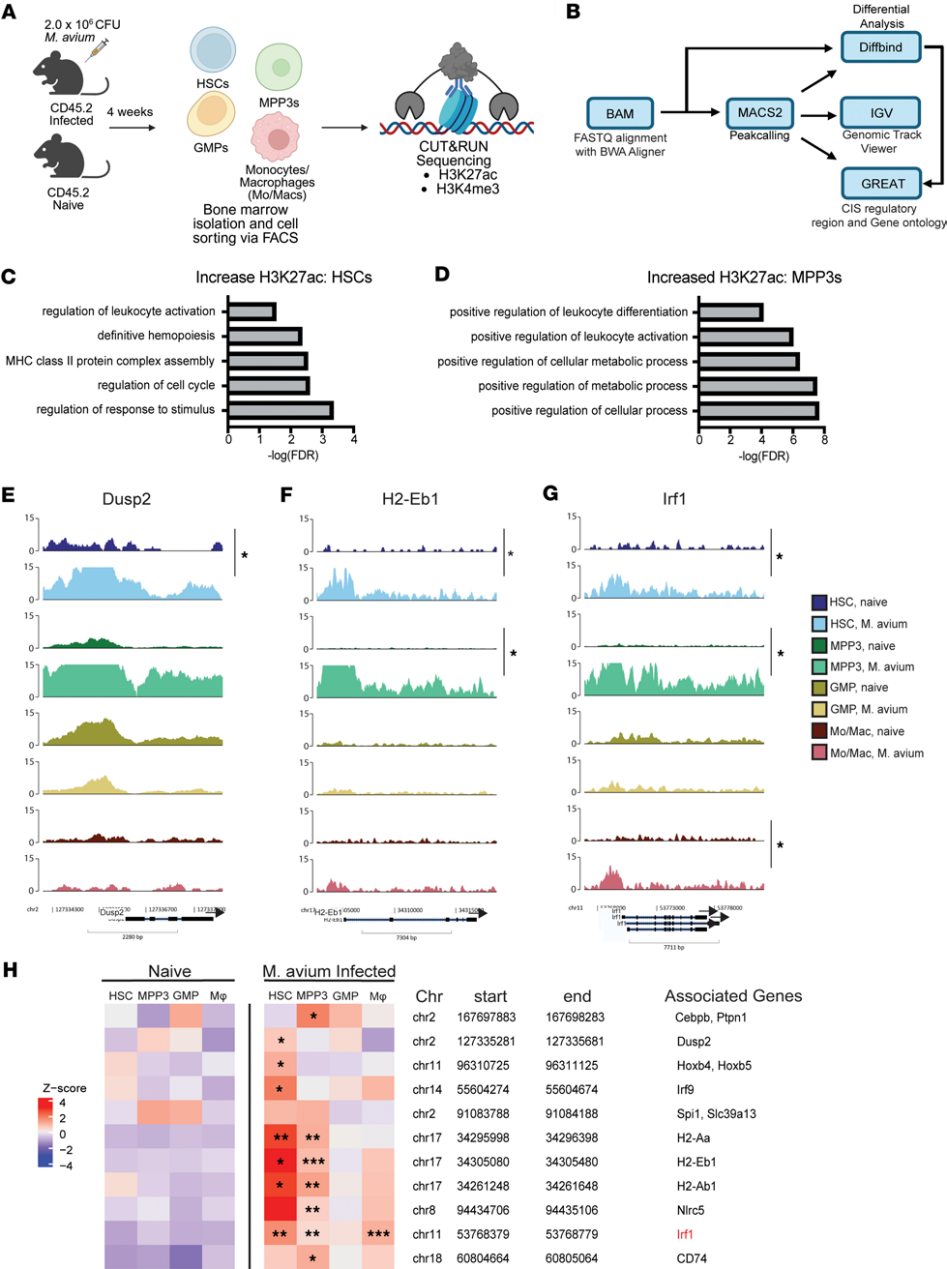
*M*. *avium–*infected HSPCs and MPP3s exhibit significant increases in H3K27ac at genomic loci associated with antigen presentation and differentiation. (**A**) Workflow of acquiring cells for cleavage under targets and release using nuclease sequencing (CUT&RUN-Seq). Experiment was performed twice. (**B**) Bioinformatic pipeline performed on CUT&RUN-generated libraries. (**C** and **D**) Gene ontology of biological processes associated with gene loci with significantly increased H3K27ac presence in HSCs (**C**) or MPP3s (**D**) from *M*. *avium–*infected mice. (**E**–**G**) Genomic tracks of H3K27ac presence at *Dusp2* (**E**), *H2-Eb1* (**F**), or *Irf1* (**G**) in HSPCs and Mo/Macs in both *M*. *avium* and naive conditions. (**H**) Histone H3K27ac enrichment heatmap of selected gene loci across *M*. *avium–*infected and naive HSPC subpopulations and macrophages, representative of 2 independent biological replicates. Associated genes in red were significant in at least 3 cell types in respective differential analyses. Bioconductor package Diffbind was used for statistical comparison of *M*. *avium* versus naive state. Cutoff FDR < 0.05 is considered significant. *P < 0.05, **P < 0.01, ***P < 0.001.

We hypothesized that changes in histone modifications are conserved through differentiation from early hematopoietic progenitors through myeloid progenitors and macrophages to mediate trained immunity. To determine if cells across 4 stages of differentiation share inflammation-induced histone modification changes, we performed cleavage under targets and release using nuclease sequencing (CUT&RUN-Seq) on cell populations from *M*. *avium–*infected and naive mice (Figure 2, A and B). We targeted HSCs, myeloid biased multipotent progenitor 3s (MPP3s), granulocyte-monocyte progenitors (GMPs), and monocytes/macrophages (Mo/Macs). H3K27ac CUT&RUN-Seq datasets we previously generated from *M*. *avium–*infected and naive HSCs were used for differential analysis (25).

Profiling of cell populations through PCA plotting and Pearson correlation (Supplemental Figure 2, A and B) showed that our cell populations clustered as expected based on cell type. Cell type was a stronger driver of cell clustering than infection, as cells from naive and infected animals were interspersed in the PCA. MPP3s and GMPs were similar, with Pearson correlation blending both populations (Supplemental Figure 2B), consistent with their shared role as myeloid-biased progenitors.

Peakcalling of the samples in both *M*. *avium–*infected and naive states revealed that all cell types had high levels of H3K27ac histone modifications across the chromatin landscape for general biological processes important for maintaining cellular function. For example, all HSPCs contained H3K27ac modifications at gene loci associated with stemness, including *Adipor2*, *Irf2bp2*, and *Spi1* (Supplemental Figure 2, C–E). Overall, H3K27ac datasets captured loci expected to be essential for hematopoietic cellular functions.

We next performed differential analysis, comparing each *M*. *avium–*infected subpopulation to their naive counterpart. These comparisons were performed to determine whether any changes seen at 1 subpopulation were consistent through differentiation. HSCs displayed a significant increase in H3K27ac modifications at regions associated with immune system processes (FDR = 7.05 × 10^–3^), definitive hemopoiesis (FDR = 6.72 × 10^–3^), and regulation of cell cycle (FDR = 4.07 × 10^–3^) (Figure 2C and Supplemental Tables 1 and 2). These included *Dusp2* (FDR = 2.3 × 10^–2^, FC = 2.29), *Irf1* (FDR = 4.2 × 10^–3^, FC = 2.62), and *Hoxb4* (FDR = 2.9 × 10^–2^, FC = 1.95) (Figure 2, E and G, Supplemental Figure 2F, and Supplemental Tables 1 and 2). Additionally, H3K27ac was increased at MHC-II antigen processing and antigen presentation genes (FDR = 1.23 × 10^–5^) like *H2-Aa* (FDR = 7.95 × 10^–4^, FC = 3.71)*, H2-Eb1* (FDR = 4.28 × 10^–4^, FC = 4.45)*,* and *H2-Ab1* (FDR = 2.5 × 10^–2^, FC = 2.01) (Figure 2F; Supplemental Figure 2, G and H; and Supplemental Tables 1 and 2). These findings are consistent with reports that HSCs harbor antigen processing and presentation capacity (36).

These changes in H3K27Ac were mirrored in MPP3s (Figure 2D). Like HSCs, MPP3s exhibited significant increases of H3K27ac at *H2-Aa* (FDR = 7.26 × 10^–3^, FC = 1.77)*, H2-Eb1* (2.75e-04, FC = 2.11)*,* and *Irf1* (FDR = 1.48 × 10^–3^, FC = 1.82) (Figure 2, F and G, Supplemental Figure 2, G–J, and Supplemental Tables 3 and 4). MPP3s uniquely displayed increases of H3K27ac at *Cebpb* (FDR = 1.14 × 10^–2^, FC = 1.58) (Supplemental Figure 2K), a pioneer factor that is critical in LPS-driven HSPC-trained immunity (18).

By comparison, GMPs showed minimal increases in H3K27ac, though many loci had high baseline acetylation (Supplemental Tables 5 and 6). Notably, Mo/Macs from *M*. *avium–*infected mice exhibited a significant increase in H3K27ac compared with naive Macs at *Irf1* (FDR = 5.35 × 10^–4^, FC = 3.73) (Figure 2G and Supplemental Table 7), a critical mediator of IFN responses in Macs (17, 37).

Collectively, we did not find any loci with significantly increased H3K27ac across all 4 stages of myeloid differentiation, indicating that H3K27ac alone cannot account for CTI conservation in myeloid progenitors (Figure 2H). However, increased H3K27ac was conserved in *M*. *avium*–infected HSCs, MPP3s, and Mo/Macs at *Irf1,* one of the few sites with increased H3K27ac across several stages of differentiation.

### HSPCs and Mo/Macs share H3K4me3 modifications at loci associated with IFN responses and GTPase function.

We next performed CUT&RUN-Seq analysis for H3K4me3 (Supplemental Figure 3, A and B). Cells clustered similarly to our H3K27ac datasets, with MPP3s and GMPs blending based on Pearson correlation (Supplemental Figure 3B).

As expected, H3K4me3 marks were found at loci associated with basic cellular function in all cell populations in both naive and *M*. *avium–*infected states, including known HSPC regulators like *Spi1/Pu.1* and *Runx1* (Supplemental Figure 3, C and D). Interestingly, many inflammatory response genes, specifically IFN signaling pathway genes *Ifnar1*, *Stat1*, and *Stat2*, and *Tet2* had steady H3K4me3 signal in all cell types and states (Supplemental Figure 3, E–I). These findings reinforce the concept that tonic levels of IFN signaling within the bone marrow contribute to steady state hematopoiesis (38–40).

**Figure 3:**
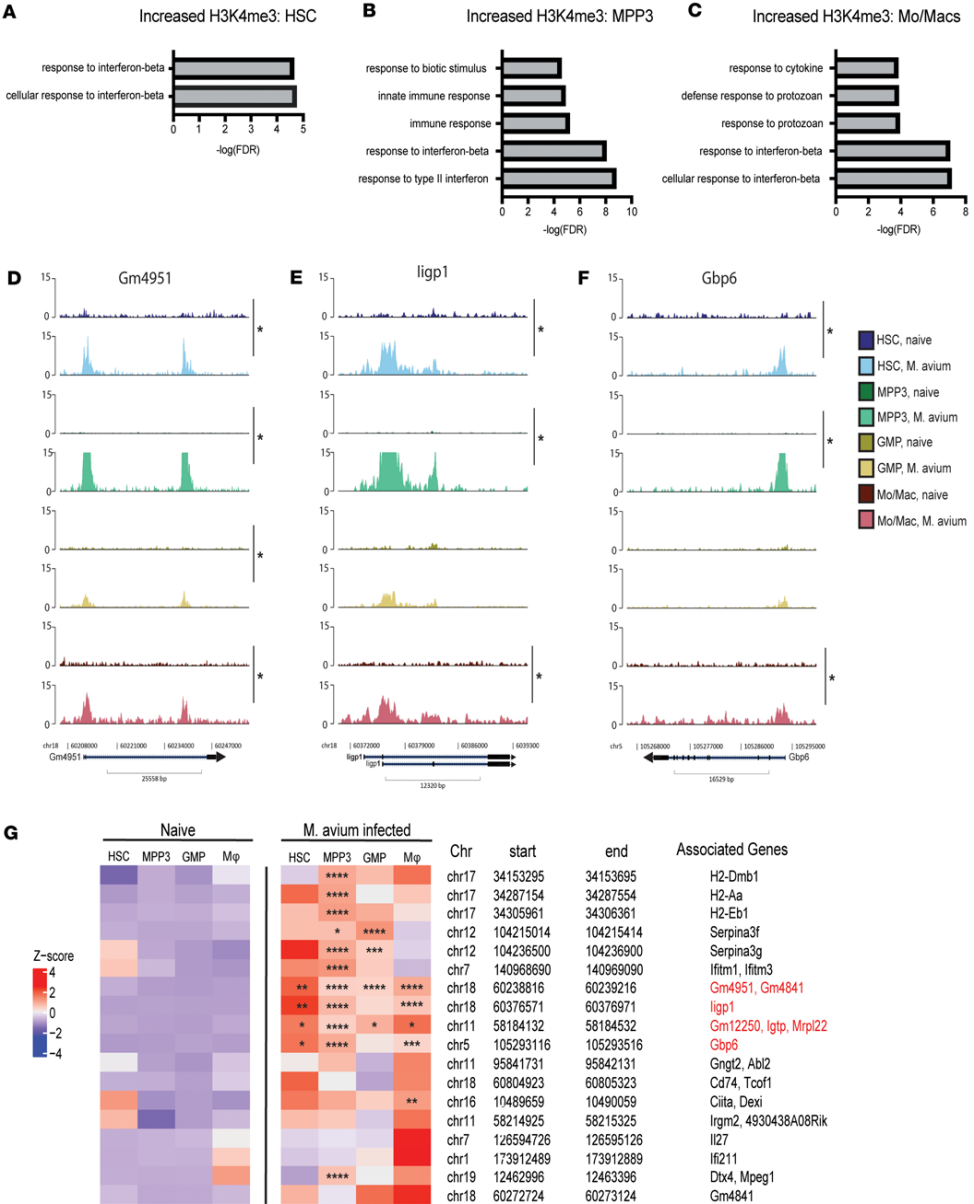
*M*. *avium–*infected HSPCs and macrophages exhibit significant increases in H3K4me3 at genomic loci associated with responses to IFN-β. (**A**–**C**) Gene ontology of biological processes associated with gene loci with significantly increased H3K4me3 presence in HSCs (**A**), MPP3s (**B**), or Mo/Macs (**C**) from *M*. *avium*–infected mice. (**D**–**F**) Genomic tracks of H3K4me3 presence at *Gm4951* (**D**), *Iigp1* (**E**), or *Gbp6* (**F**) in HSPCS and Mo/Macs in both *M*. *avium–*infected and naive conditions. (**G**) Histone H3K4me3 enrichment heatmap of selected gene loci across *M*. *avium–*infected and naive HSPC subpopulations and macrophages, representative of 2 biological replicates. Associated genes in red were significant in at least 3 cell types in respective differential analyses. Data compiled from 2 independent experiments. Bioconductor package Diffbind was used for statistical comparison of *M*. *avium* versus naive state. Cutoff FDR < 0.05 is considered significant. *P < 0.05, **P < 0.01, ***P < 0.001, ****P < 0.0001.

In HSCs, there were significant increases in H3K4me3 at biological processes associated with responses to IFN-β (FDR = 9.69 × 10^–6^) (Figure 3A and Supplemental Tables 8 and 9). Many of these enriched loci were closely tied to GTPase activity (FDR = 8.89 × 10^–3^) and GTP binding (FDR = 1.59 × 10^–2^) (Supplemental Tables 8 and 9), including *Gm12250* (FDR = 2.3 × 10^–2^, FC = 1.82), *Gm4951* (FDR = 5.64 × 10^–3^, FC = 2.91), *Iigp1* (FDR = 1.88 × 10^–3^, FC = 3.83), and guanylate binding protein 6 (*Gbp6*) (FDR = 2.32 × 10^–2^, FC = 2.72) (Figure 3, D–G, Supplemental Figure 3J, and Supplemental Tables 8 and 9). While several of these GTPases are mouse specific, GBP6 and GBP family proteins have human homologs that are reported to be crucial in mycobacterial immunity (41, 42).

Much like HSCs, MPP3s from *M*. *avium–*infected mice had significant increases in H3K4me3 at the same set of GTPases (Figure 3, D–G; Supplemental Figure 3D; and Supplemental Tables 10 and 11) and immune processes including responses to IFN-β (FDR = 1.82 × 10^–11^), type II IFN (FDR = 3.36 × 10^–6^), and innate immune responses (FDR = 3.71 × 10^–3^) (Figure 3B and Supplemental Tables 10 and 11). Immune response genes such as *Ifitm1* (FDR = 2.36 × 10^–6^, FC = 2.44), *Batf2* (FDR = 4.33 × 10^–3^, FC = 2.55), *Serpina3f* (FDR = 3.24 × 10^–2^, FC = 1.44), and *Serpina3g* (FDR = 6.27 × 10^–8^, FC = 2.25) were also significantly increased upon *M*. *avium* infection (Figure 3G; Supplemental Figure 3, K–M; and Supplemental Tables 10 and 11). GMPs from *M*. *avium*–infected mice showed increased H3K4me3 modification at *Gm4951* (FDR = 9.71 × 10^–8^, FC = 3.99), *Serpina3f* (FDR = 1.06 × 10^–5^, FC = 3.78)*,* and *Serpina3g* (FDR = 7.63 × 10^–4^, FC = 1.75), while H3K4me3 modification was unchanged at *Gbp6* and *Iigp1* (Figure 3, D and G; Supplemental Figure 3M; and Supplemental Tables 12 and 13). Like H3K27ac, the H3K4me3 mark was present at baseline in GMPs at many of the sites at which we found increases in other cell populations, reducing the sensitivity of the differential analysis in this cell type (Supplemental Tables 12 and 14).

Mo/Macs from *M*. *avium–*infected mice also showed consistent increases in H3K4me3 associated with responses to IFN-β (FDR = 2.1 × 10^–8^) (Figure 3C and Supplemental Tables 15 and 16) and at GTPases like *Gm4951* (FDR = 4.62 × 10^–5^, FC = 2.98), *Iigp1* (FDR = 6.07 × 10^–5^, FC = 2.99), and *Gbp6* (FDR = 1.56 × 10^–4^, FC = 2.63) (Figure 3, D–G). *Ciita* (FDR = 3.83 × 10^–3^, FC = 1.58), a master transcription factor (TF) of MHC-II antigen presentation, showed increased H3K4me3 in this analysis (43) (Supplemental Figure 3N and Supplemental Tables 15 and 16). Interestingly, H3K4me3 marks were decreased in Mo/Macs upon infection at some gene loci associated with metabolism, DNA repair, and macromolecule processing (Supplemental Tables 15 and 17), while other regions such as IL-27 (*Il-27*), a multifunction cytokine that can potentially elicit Th1 responses, were unchanged (44) (Figure 3G and Supplemental Figure 3O).

Overall, we discovered a limited number of loci with increased H3K4me3 conserved across all 4 stages of myeloid differentiation upon *M*. *avium* infection, including GTPases *Gm4951* and *Gbp6* that mediate IFN-β responses (Figure 3G). To determine whether these findings were specific to *M*. *avium* infection, we tested whether H3K4me3 marks were induced at *Gbp6* after BCG exposure. CUT&RUN qPCR of Gbp6 showed similar acquisition of H3K4me3 after either *M. avium* or BCG infection, indicating a common response to both training agents (Supplemental Figure 3P).

### Loci associated with IFN responses and GTPase function showed increased chromatin accessibility in HSPCs and Mo/Macs upon M. avium infection.

**Figure 4:**
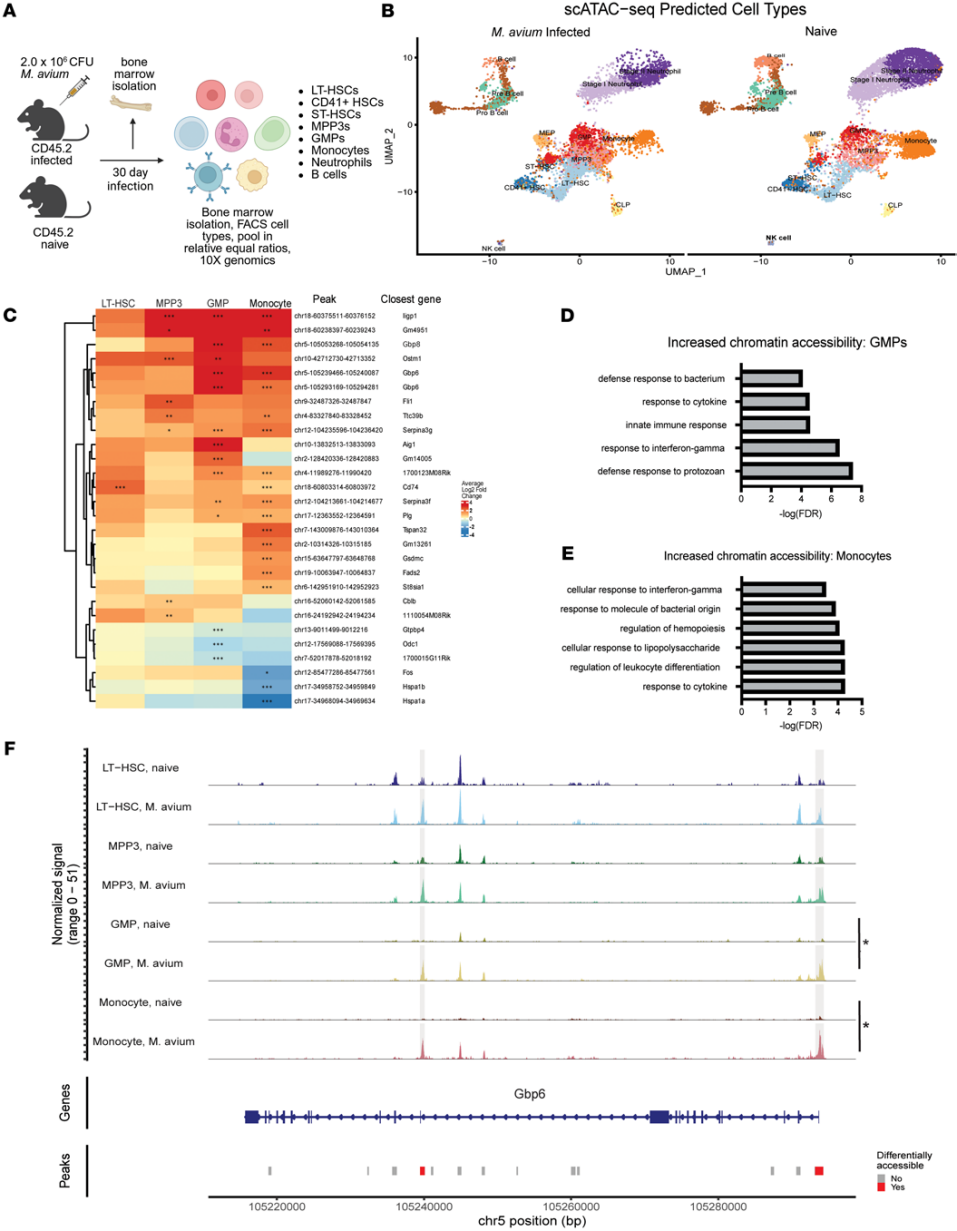
HSPCs and monocytes exhibit distinct chromatin accessibility changes upon *M*. *avium* infection. (**A**) Workflow schematic for acquiring targeted cell populations for scATAC-Seq. (**B**) Predicted UMAP plotting of targeted HSPC and lineage subpopulations in both naive and *M. avium*–infected states. (**C**) Heatmap accessibility of peaks found to be significantly changed in at least 1 cell type, with asterisks representing the level of significance at each gene locus in a specific cell population. (**D** and **E**) Gene ontology enrichment analysis in GMPs (**D**) and monocytes (**E**). (**F**) Genomic coverage plot of Gbp6. Cell populations with significant increases were marked with an asterisk. Peaks that are significantly differentially accessible (*P*_adj_ < 0.05) between *M. avium*–infected and naive states in at least 1 cell type are highlighted with an asterisk.

To understand how the overall chromatin accessibility landscape is changed in HSPCs upon *M*. *avium* infection, we performed single-cell assay for transposase accessibility sequencing (scATAC-Seq) on targeted HSPCs and downstream terminally differentiated cells, including Mo/Macs, neutrophils, and B cells, from pooled mouse samples (Figure 4A and Supplemental Figure 4A). To mirror our prior single-cell transcriptomic study using the same infection model (22), we specifically isolated long-term HSCs (LT-HSCs) and myeloid-biased subpopulations, including CD41^+^ HSCs, MPP3s, and GMPs, to acquire sufficient cell numbers for differential analyses and enhance representation of rare cell types. We found similar clustering in both *M*. *avium–*infected and naive states, with HSCs near MPPs and GMPs clustering near Mo/Macs subpopulations (Figure 4B). Neutrophils were comparatively distant from the HSPC clusters, indicating a more distinct chromatin landscape. Like our CUT&RUN-Seq datasets, GMPs and MPP3s blended together in the predicted UMAPs. Interestingly, we saw a significant separation between common lymphoid progenitors (CLPs) and B cells, suggesting that the chromatin landscape of lymphoid cells changes extensively during terminal differentiation.

Peakcalling revealed chromatin regions that were consistently accessible across progenitors and differentiated myeloid cells independent of *M*. *avium* infection. These included regions such as *Jun* and *Irf1,* both TFs we have previously reported in HSPC transcriptional responses to *M*. *avium* infection (17) (Supplemental Figure 4, B and C). These data indicate that the chromatin is already open at these loci, with induction of transcription depending more on additional factors such as TFs or acquisition of active histone modifications (17, 18).

We next performed differential analysis to identify chromatin accessibility changes upon *M*. *avium* infection. Gene ontogeny analysis revealed that no biological processes were significantly more accessible in *M*. *avium–*infected LT-HSCs (Supplemental Table 18). However, the antigen presentation gene *Cd74* had increased accessibility in LT-HSCs upon *M*. *avium* infection (*P*_adj_ = 2.04 × 10^–9^, log_2_FC ~2.17) (Supplemental Figure 4D and Supplemental Table 18). *M*. *avium–*infected MPP3s exhibited changes in chromatin accessibility at genomic loci identified in our CUT&RUN-Seq analysis (Supplemental Table 19). These included *Iigp1* (*P*_adj_ = 3.24 × 10^–7^, log_2_FC ~4.23)*, Serpina3g* (*P*_adj_ = 1.18 × 10^–2^, log_2_FC ~0.88)*, Gm4951* (*P*_adj_ = 1.16 × 10^–2^, log_2_FC ~4.50)*,* and *Gm4841* (*P*_adj_ = 4.66 × 10^–2^, log_2_FC ~3.59) (Figure 4C, Supplemental Figure 4, E–G, and Supplemental Table 19).

Comparatively, we found many more chromatin accessibility changes in GMPs and Mo/Macs upon *M*. *avium* infection. IFN-associated biological responses such as response to protozoan and bacterium and response to cytokines were increased in GMPs upon infection (Figure 4D and Supplemental Table 20). GTPases that we previously noted including GBPs; *Gbp6* (*P*_adj_ = 3.24 × 10^–9^, log_2_FC = 3.41), *Gbp8* (*P*_adj_ = 6.47 × 10^–6^, log_2_FC = 4.55), *Iigp1* (*P*_adj_ = 4.26 × 10^–5^, log_2_FC = 9.26), *Irgm2* (*P*_adj_ = 2.20 × 10^–4^, log_2_FC ~1.59), *Serpina3g* (*P*_adj_ = 6.92 × 10^–6^, log_2_FC = 1.41), and *Serpina3f* (*P*_adj_ = 5.03 × 10^–3^, log_2_FC = 1.12) had significantly increased chromatin accessibility in GMPs from *M*. *avium*–infected mice (Figures 4, C and F, Supplemental Figure 4F, and Supplemental Table 20). Many of these loci demonstrated histone modifications at the MPP3 level in the CUT&RUN-Seq, leaving open the possibility that epigenetic changes occurring at those loci may have contributed to chromatin openness at the downstream GMP level.

Significant changes in chromatin accessibility were seen in Mo/Macs from *M*. *avium*–infected mice at loci similar to GMPs, including *Gbp6* (*P*_adj_ = 5.57 × 10^–10^, log_2_FC = 1.95), *Gbp8* (*P*_adj_ = 2.95 × 10^–10^, log_2_FC = 2.46)*, Serpina3g* (*P*_adj_ = 6.15 × 10^–14^, log_2_FC = 1.95), and *Serpina3f* (*P*_adj_ = 2.00 × 10^–8^, log_2_FC ~1.37) (Figure 4, C and F, Supplemental Figure 4G, and Supplemental Table 21). Additional GBPs — *Gbp7* and *Gbp2* (*P*_adj_ = 2.62 × 10^–3^, log_2_FC ~0.966), *Gbp9* and *Gbp4* (*P*_adj_ = 1.17 × 10^–2^, log_2_FC = 0.759), and secreted factors (*Il12a* (*P*_adj_ = 3.69 × 10^–4^, log_2_FC ~0.324), *Il12b* (*P*_adj_ = 1.299 × 10^–6^, log_2_FC = 1.08), *Il9* (*P*_adj_ = 4.43 × 10^–3^, log_2_FC = 1.09) — were also increased (Supplemental Table 21). We also found *Ccl5,* a chemokine found associated with COVID-19, HIV, and HSC IFN responses (25, 26, 45), had increased in accessibility in Mo/Macs (*P*_adj_ = 3.72 × 10^–6^, log_2_FC = 0.557) (Supplemental Table 21). Gene set enrichment analysis indicated that biological processes associated with immune responses, including response to cytokine and responses to IFN-γ were significantly more accessible (Figure 4E and Supplemental Table 21). The inflammatory gene set “response to lipopolysaccharide,” including loci such as at *Cebpe* (*P*_adj_ = 8.39 × 10^–4^, log_2_FC = 0.764), *Icam1* (*P*_adj_ = 9.53 × 10^–3^, log_2_FC = 0.502), *Nfkbiz* (*P*_adj_ = 2.91 × 10^–9^, log_2_FC ~1.58), *Tnfaip3* (*P*_adj_ = 1.96 × 10^–3^, log_2_FC = 1.01), and *Tnfrsf1b* (*P*_adj_ = 2.95 × 10^–3^, log_2_FC = 0.342) was also enriched (Figure 4E and Supplemental Table 21). Antigen processing genes *Cd74* (*P*_adj_ = 5.89 × 10^–8^, log_2_FC = 0.586) and *H2-Aa* (*P*_adj_ = 5.36 × 10^–3^, log_2_FC = 0.807) were also enriched in Mo/Macs (Supplemental Figure 4, D and G, and Supplemental Table 21). Overall, these findings affirm that Mo/Macs undergo changes in the chromatin landscape to elicit inflammation-mediated immune responses.

Collectively, these findings reveal only a few sites with consistently open or increased chromatin accessibility across 4 stages of myeloid differentiation. Notably, IFN-stimulated TFs like *Jun* and *Irf1* had consistent accessibility, while GTPases like *Gm4951*, which was significant in MPP3s and Mo/Macs, had partial increases in all HSCs and GMPs without reaching statistical significance (Supplemental Figure 4E). *Gbp6*, a locus that demonstrated increased H3K4me3 across several cell stages, gained chromatin accessibility in HSPCs, GMPs, and Mo/Macs (Figure 4F). Consistent with the CUT&RUN data, these areas of conserved increased chromatin accessibility lay in IFN and immune response genes.

### HSPC subpopulations and Mo/Macs from M. avium–infected mice have conserved enrichment of motifs associated with IFN and NF-κB signaling.

TFs are DNA-binding proteins known to participate in coactivator/corepressor recruitment, transcription complex formation initiation, DNA looping, polymerase II recruitment, and — more recently — pioneer factors that initiate and maintain chromatin openness (46–48). Studies have shown that TFs may play a pivotal role in the epigenetic memory of various long-living cell populations, including skin and blood stem cells (18, 49, 50)^.^ Notably, CEBPβ has been reported as a pioneer factor crucial for promoting transcription and chromatin accessibility of genes associated with LPS-stimulated HSPC trained immunity (18). Given these findings, we evaluated our scATAC-Seq dataset to determine how *M*. *avium* infection affects TF function across stages of myeloid differentiation.

**Figure 5:**
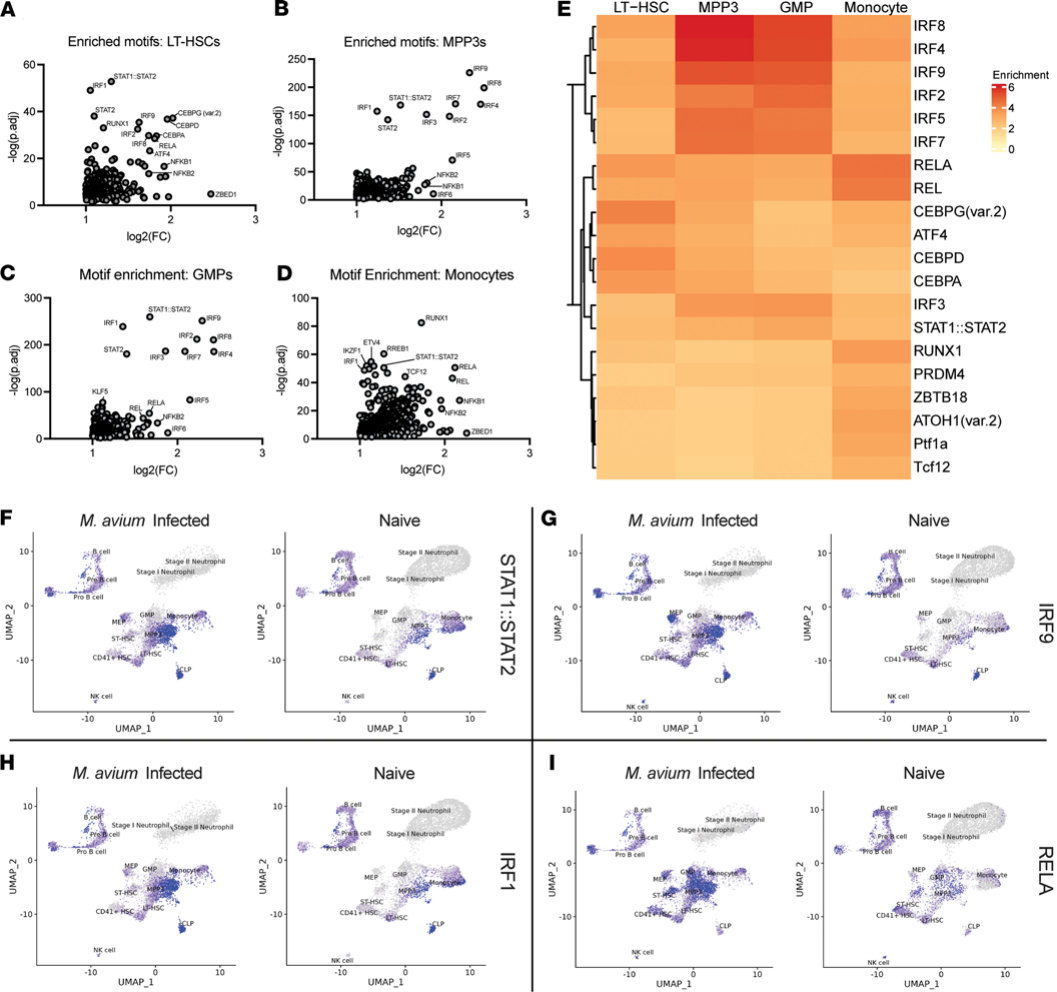
*M*. *avium–*infected HSPC subpopulations and monocytes have a conserved, increased enrichment of motifs associated with IFN and NF-κB signaling. (**A**–**D**) Volcano plot of motifs significantly enriched in *M*. *avium*–infected LT-HSCs (**A**), MPP3s (**B**), GMPs (**C**), and monocytes (**D**) versus naive controls. Motifs identified based on scATAC-Seq datasets. (**E**) Heatmap showing the highest average log fold enriched motifs across *M*. *avium–*infected LT-HSCs, MPP3s, GMPs, and macrophages. Cutoffs for filtering included *P* < 1 × 10^–20^ and LFC > 2 observed in any cell type. (**F**–**I**) Motif enrichment UMAP plots of STAT1::STAT2 (**F**), IRF9 (**G**), IRF1 (**H**), and RELA (**I**) in infected and naive states of HSPC subpopulations. Volcano plots (**A**–**D**) were generated for significantly enriched motifs with *P*_adj_ < 0.05 and FC > 1.

We found a significant increase of inflammatory TF motifs across all cell populations upon *M*. *avium* infection. Among HSCs, we expected to find IRF and STAT signaling motifs, as mycobacterial infections strongly induce IFN signaling in HSPCs (17, 21, 24, 51, 52). Indeed, HSCs from *M*. *avium–*infected mice were enriched in inflammatory motif signatures associated with IFN signaling — STAT1::STAT2 (*P*_adj_ = 1.45 × 10^–3^, FC = 2.46), IRF1 (*P*_adj_ = 8.22 × 10^–50^, FC = 2.07), STAT2 (*P*_adj_ = 8.09 × 10^–39^, FC = 2.14), IRF9 (*P*_adj_ = 3.90 × 10^–36^, FC = 3.09), IRF8 (*P*_adj_ = 1.60 × 10^–30^, FC = 3.34). IRF9 and STAT1/2 factors are known to combine to create the stimulated gene factor 3 (ISGF3) complex for canonical type I IFN signaling, and this paradigm is supported by our H3K4me3 CUT&RUN differential analysis. Additional binding motifs associated with NF-κB signaling — RELA (*P*_adj_ = 3.03 × 10^–29^, FC = 3.50), NFKB1 (*P*_adj_ = 2.43 × 10^–17^, FC = 3.78), NFKB2 (*P*_adj_ = 3.03 × 10^–14^, FC = 3.34), CEBP signaling (CEBPG (var.2) (*P*_adj_ =6.17 × 10^–38^, FC = 4.07), and CEBPD (*P*_adj_ = 1.63 × 10^–37^, FC = 3.89) — and master regulator Runx1 (*P*_adj_ = 8.61 × 10^–34^, FC = 2.31) were all enriched in HSCs from infected mice (Figure 5A and Supplemental Table 22). These findings were not unexpected, given that NF-κB and CEBP can be activated upon IFN signaling via PI3K/Akt signaling and MAPK signaling, respectively (53–55). Independently, NF-κB signaling via TNF-α has been recently reported to activate IRF1 and IRF3 (56), further highlighting an interconnection between IRFs and NF-κB activity.

These sets of TF family motifs were strongly enriched in myeloid progenitors and Mo/Macs from *M*. *avium*–infected mice. MPP3s and GMPs exhibited significant enrichment of IRFs, NF-κBs, and CEBP families upon infection (Figure 5, B and C, and Supplemental Tables 23 and 24). Among Mo/Macs, the strongest enrichment occurred in NF-κB motifs, including NFKB1 (*P*_adj_ = 3.91 × 10^–28^, FC = 4.52), NFKB2 (*P*_adj_ = 4.32 × 10^–22^, FC = 3.90), RELA (*P*_adj_ = 2.25 × 10^–51^, FC = 4.35), and REL (*P*_adj_ = 6.90 × 10^–44^, FC = 4.26), though IRF and CEBP families were also enriched (Figure 5, D and E, and Supplemental Table 25). Collectively, IRF, CEBP, and NF-κB TF families were some of the most enriched motifs across HSPCs, GMPs, and Mo/Macs, suggesting that the activity of these TFs could play a prominent role in CTI (Figure 5, E–I, and Supplemental Tables 22–25). This conserved enrichment could only be observed through Mo/Mac differentiation, whereas neutrophils showed little enrichment of these motifs upon *M*. *avium* infection. Separately, these motifs were constitutively present in lymphoid cell populations (Figure 5, F–H). Thus, TF motif analysis affirms strong conservation of IFN, CEBP, and NF-κB signaling among HSPCs, GMPs, and Mo/Macs.

### H3K4me3 modifications acquired upon M. avium training are transient.

**Figure 6:**
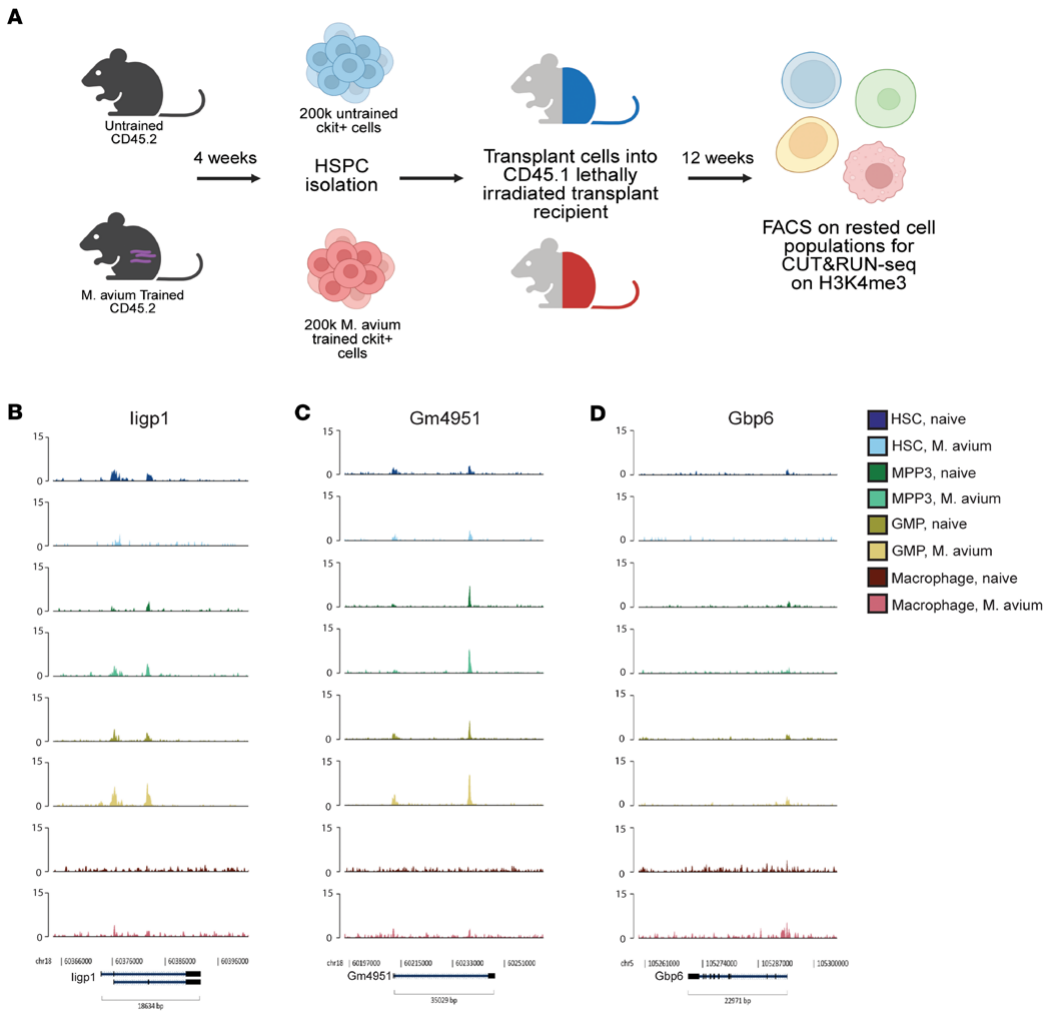
Histone modification H3K4me3 acquired in *M*. *avium* training do not persist in HSPCs that are transplanted and rested. (**A**) Workflow of performing CUT&RUN on H3K4me3 in HSPCs and Mo/Macs that had been rested and transplanted into lethally irradiated recipients. In total, 200,000 CD45.2 M. avium–trained or untrained HSPCs (cKit^+^) were transplanted with 200,000 CD45.1 rescue marrow into lethally irradiated recipients. (**B**–**D**) Genomic tracks of Iigp1 (**B**), Gm4951 (**C**), and Gbp6 (**D**) in either untrained, rested or *M. avium*–trained, rested HSPCs, and Mo/Macs acquired from transplanted recipients. Data compiled from 2 independent experiments. MACS2 and Diffbind were utilized for analysis.

Given that *M*. *avium–*trained HSPCs, myeloid progenitors, and Mo/Macs acquired highly conserved H3K4me3 modifications and increased chromatin accessibility at loci associated with IFN-β, we asked whether this histone mark remained stable in rested, *M*. *avium–*trained HSPCs. To answer this question, we transplanted *M*. *avium–*trained or untrained HSPCs into lethally irradiated CD45.1 mice and waited 12 weeks to assess the H3K4me3 modification landscape (Figure 6A). This transplant method removes the HSPCs and Mo/Macs from the infected environment and provides sufficient time for them to return to steady state. As shown in Figure 1, donor BMDMs from similarly transplanted recipients retain persistent functional changes and support improved bacterial clearance, indicating CTI. Upon CUT&RUN-Seq, we found that H3K4me3 did not persist in progenitor or downstream Mo/Mac populations (Figure 6A). IFN-β associated loci including *Iigp1*, *Gm4591*, and *Gbp6* showed little to no H3K4me3 presence in both untrained and *M*. *avium–*trained, rested states (Figure 6, B–D). Thus, while our findings point toward IFN-β–associated loci as key mechanistic components for trained immunity phenotypes, absence of H3K4me3 in the resting state indicates that this is not the relevant epigenetic mark to encode this memory.

### Loss of GBP6 affects metabolic functions of PMA-treated THP-1 Macs, while loss of IRF1 negatively affects both metabolic and immune function.

**Figure 7:**
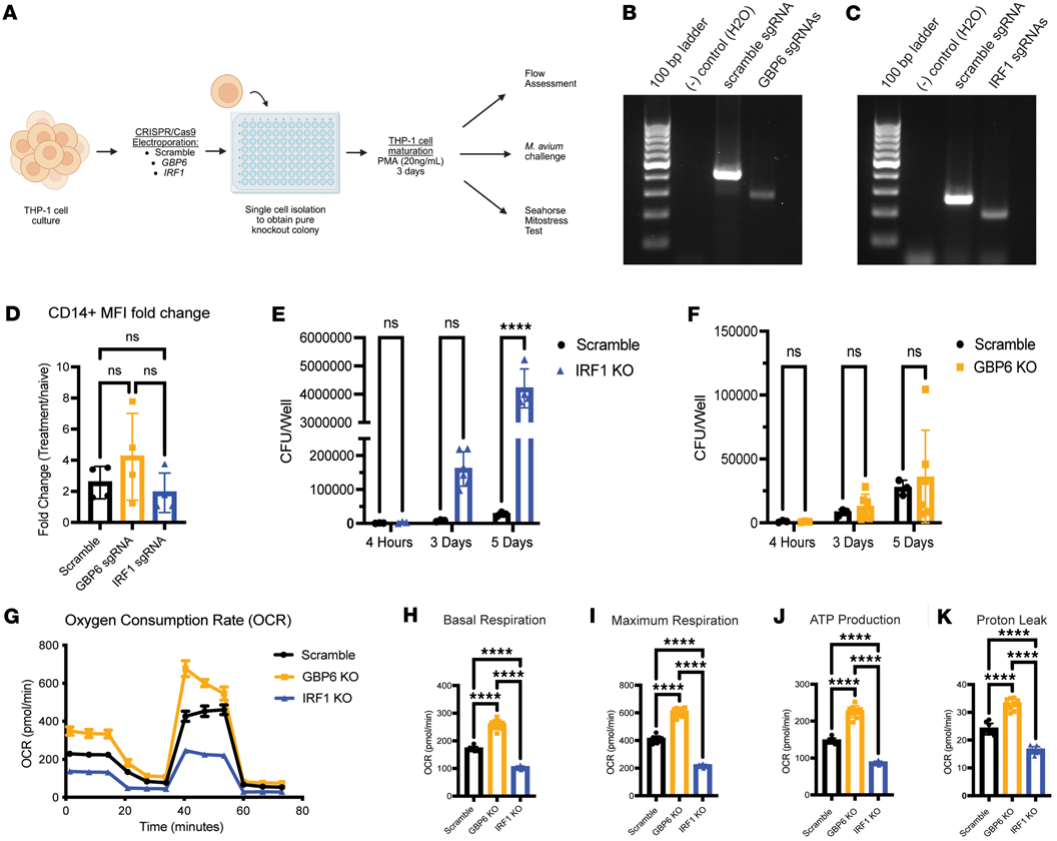
Loss of GBP6 affects cellular respiration in THP-1–KO macrophages independent of innate immune function, while loss of IRF1 negatively affects many macrophage functions. (**A**) Workflow of knocking out *GBP6* and *IRF1* in THP-1 cells, followed by their maturation into macrophages for immunophenotyping. KO colonies were derived from single CRISPR-edited cells for functional assessment. (**B** and **C**) Representative gel image of exon 2 of *GBP6* (**B**) and exon 4 of *IRF1* (**C**). (**D**) Fold change of CD14 mean fluorescence intensity upon 3-day PMA-treatment (20 ng/mL), compared with uninduced. *n* = 4 independent experiments. (**E** and **F**) Bacterial CFU from *M*. *avium–*challenged THP-1 cells (100,000 THP-1 cells per well, *M*. *avium* MOI 1) for 4 hours, 3 days, or 5 days. *IRF1* KO versus scramble control (**E**) and *GBP6-*KO versus scramble control (**F**). *n* = 3–6 per group at each time point. (**G**) Seahorse mitostress assay of Scramble PMA-treated scramble (black), *GBP6-*KO (gold), and *IRF1-*KO (blue) THP-1 cells. (**H**–**K**) Calculated basal respiration (**H**), maximum respiration (**I**), ATP production (**J**), and proton leak (**K**) of PMA-treated THP-1 cells. All experiments were performed twice independently. Two-way ANOVA with Šídák’s multiple comparisons was used for **D** and **E**, while 1-way ANOVA was used for **H**–**K**. ****P < 0.0001.

Our findings highlight that modifications or chromatin openness at loci related to IFN signaling, specifically at *Irf1* and *Gbp6*, are conserved across myeloid differentiation and may, thus, contribute directly to CTI. To assess the functional role of these 2 proteins in human Mo function, we utilized CRISPR-Cas9 editing to excise catalytic domains of *GBP6* and *IRF1*, respectively, from THP-1 cells, a human monocytic cell line (Figure 7A). Deletion of *GBP6* or *IRF1* was confirmed by PCR in single-cell–derived colonies (Figures 7, B and C), and KO cell lines were expanded for phenotypic testing (57). Both *GBP6-*KO and *IRF1*-KO cells successfully differentiated, with consistent total cell numbers, viability, and acquisition of CD14, a marker of mature human Macs (57) (Figure 7D and Supplemental Figure 5, A and B). These findings indicate that KO of *IRF1* or *GBP6* does not affect Mo-to-Mac maturation or overall viability.

Next, we challenged KO cell lines with *M*. *avium* to determine whether loss of GBP6 or IRF1 would affect bacterial killing function. Compared with controls, *M*. *avium* growth was significantly increased in challenged *IRF1-*KO wells by the day 5 time point (Figure 7E), suggesting an impaired bacterial killing capacity. In contrast, we found no difference in bacterial growth in challenged *GBP6-*KO cells compared with scramble controls at all time points (Figure 7F).

We then asked whether the loss of GBP6 and IRF1 affected mitochondrial respiration, a key factor in innate immune responses and trained immunity (4, 58). Utilizing the seahorse mitostress test on PMA-treated THP-1 cells (Figure 7F), we found that baseline and maximum respiration of *IRF1-*KO cells were significantly decreased compared with controls (Figure 7, G and H), indicating impaired oxidative phosphorylation. Furthermore, *IRF1*-KO cells also demonstrated a significant decrease in ATP production (Figure 7I). Notably, *IRF1-*KO cells had a subtle but significant decrease in proton leak compared with controls, suggesting that the decreased respiration is not due to damaged mitochondria (Figure 7J). In contrast, *GBP6-*KO cells had significantly increased respiration across all parameters measured (Figure 7, H–J). However, proton leak was also significantly increased in *GBP6-*KO cells, indicating mitochondrial damage that may be causing dysregulated metabolic function (Figure 7K). Together, these KO studies highlight that IRF1 has an essential role in Mac killing capacity and metabolism, while GBP6 plays a regulatory role in mitochondrial respiration.

## Discussion

CTI is a recently described phenomenon in which durable cell autonomous changes in HSPC lead to the generation of downstream innate immune cells with increased metabolism, cytokine production, and pathogen killing capacity. While many studies indicate that CTI enhances host immunity (18, 21, 22, 59), it also can be maladaptive, driving autoinflammation in conditions like long COVID (26, 60–63). The cell-autonomous nature of this phenomenon has been demonstrated through transplant studies and human epidemiologic studies (10, 11, 18); however, the mechanisms by which CTI is encoded within HSPCs remain unclear. Improving our understanding of how CTI develops is a critical advance to determine whether it can be manipulated to either promote host immunity or stop its maladaptive consequences. Here we evaluated whether chromatin modifications, such as histone modifications, chromatin accessibility, and TF motif enrichment, are conserved through stages of innate immune cell differentiation after infection, thereby pinpointing key mechanistic mediators of innate immune memory.

Using an established model of *M*. *avium* infection, we validated that immune training confers cell autonomous changes on HSPCs, leading to the generation of BMDMs with heightened inflammatory responses. Our findings are consistent with prior work showing that mycobacterial training produces HSPCs that provide protection against subsequent infectious challenges and can be cross-protective against antigenically distinct infections, arguing against an adaptive immune mechanism (8, 18, 22, 59).

Both CUT&RUN analysis of H3K4me3 and single-cell ATAC-Seq analysis demonstrated that IFN signaling was significantly increased in *M*. *avium–*trained HSPCs, MPP3s, GMPs, and Mo/Macs. Notably, the strongest responses were found in the myeloid progenitors, similar to what has been demonstrated after BCG training in humans (19). Furthermore, motif enrichment analysis revealed IRFs to be some of the highest ranked enriched motifs across the cell types. Inflammatory motifs representing NF-κB signaling and CEBP signaling, which can be noncanonically activated by IFNs (18, 54, 55, 64, 65), were also highly enriched. Notably, NF-κB, IRF1, and ISGF3 form a network of TFs that has been reported in LPS and IFN-γ training of BMDMs to contribute to the development of H3K4me1 de novo superenhancers (56). This motif network is found not only in mouse models but also in patients recovered from SARS-CoV-2, which also show a consistent increase in IRF and NF-κB binding motifs (26). Additionally, CEBP and IRF TFs are highly enriched in unique myeloproliferative neoplasm subpopulations that emerge upon type I IFN therapeutics (66). Our findings, summarized collectively in Table 1, further support a critical role for this conserved signaling network in CTI.

With CUT&RUN-Seq, we determined the histone modification landscape of H3K27ac and H3K4me3 in HSPCs and Mo/Macs in both naive and *M*. *avium–*infected states. Recent studies have suggested that H3K27ac and H3K4me3 marks critically prime key CTI loci at the progenitor level (18, 67) and that these marks are passed to downstream effector Mo/Macs to produce trained immune memory. However, our data do not strongly support this hypothesis, as we find that most H3K4me3 marks do not persist in the resting stage after infection, and H3K27ac marks are known to be very short-lived (68). Other forms of epigenetic modification or stable TF networks may support CTI. For example, a study reported the role of lactylation as a durable, novel modification critical for the generation and persistence of CTI in BCG-vaccinated reprogramming (67). Assessment of this and other durable marks at sites we identified through scATAC-Seq analysis will likely improve our understanding of the structural basis of CTI.

Increased H3K27ac was conserved at *Irf1* across HSCs, MPP3s, and Mo/Macs upon *M*. *avium* infection, and the IRF1 binding motif was also highly enriched across all 4 cell types upon infection. While H3K27ac tends to be a transient marker that persists for less than a day (68), the active production of IRF1 during *M*. *avium* infection (17) and its high level of motif availability indicates IRF1 as a central mediator of inflammatory responses relevant to CTI. Prior studies have demonstrated that TFs including CEBPβ (18) in HSPCs and AP1 TFs in epithelial skin stem cells and fibroblasts (69, 70) can serve as critical pioneer factors in trained immunity. IRF1 is essential for both HSPC inflammatory–induced differentiation responses (17, 26, 71, 72) and Mac responses involved in IFN responses and innate immune function responses to viral and bacterial responses (37, 71, 73–76). Much as CEBPβ plays a multifunctional role in processing and relaying inflammatory signaling, we believe that IRF1 also serves as a central hub that relays many inflammatory signals to elicit the innate immune response. Indeed, we found that CRISPR KO of *IRF1* severely impairs human THP1 Mo and Mac functions, consistent with prior reports of inborn errors of immunity (IEI) (37, 74). Given IRF1’s critical role in promoting gene transcription of metabolic and inflammatory pathways, including production of TNF-α, we speculate that loss of IRF1 function severely dampens the inflammatory reprogramming of HSPCs necessary for CTI (77). Notably, Moorlag et al. reported increased chromatin accessibility at other IRF family members, notably IRF5 and IRF8, in human Mos after BCG vaccination (78), highlighting the relevance of this family.

Consistent with the IFN theme, we found conserved acquisition of H3K4me3 in genes associated with biological responses to IFN-β. GTPases such as *Gm4951*, *Iigp1*, *Gm4841*, and *Gbp6* were prominent among these genes. While many of these GTPases are found only in mice, guanylate binding proteins, including GBP6, are reported to be essential for immunity in both mice and humans. For example, silencing of GBP6/GBP10 decreases immune responses in Macs challenged with *Mycobacterium bovis* (41, 42, 79–81). Notably, GBP4 and GBP5 confer transcriptional memory in HeLa cells responding to IFN-γ, suggesting that GBP family proteins play a role in inflammation-induced immune memory (82, 83). In our scATAC-Seq analysis, *Gbp6* gained increased chromatin accessibility across HSPCs and Mo/Mac cell stages. GBP6-KO studies revealed that loss of *GBP6* affected metabolic respiration but not bacterial killing, in contrast to prior studies (42, 79). Specifically, we found an overall increase in mitochondrial respiration in *GBP6-*KO THP1 like Macs, possibly due to higher demands on oxidative phosphorylation at the expense of mitochondrial integrity. We speculate that this overall increase in mitochondrial respiration may be a compensatory mechanism for the damaged mitochondria in *GBP6-*KO cells. Our findings indicate a possible regulatory role for GBP6 in mitochondrial respiration independent of intracellular pathogen responses. These findings set the stage for future studies on the role of GBP6 in Mac function and CTI (84). Interestingly, GBP6 has been reported to be transcriptionally regulated by IRF1, linking regulation of these 2 key genes (74).

### Limitations of the study

In this study, we focused our CUT&RUN-Seq on 2 common histone modifications, H3K27ac and H3K4me3, which have been speculated to contribute to CTI in prior studies. However, many other chromatin modifications, both silencing and activating, may affect gene transcription. Careful examination of other marks, guided by the targets identified in this study, will be important in future studies. In scATAC-Seq, it should be noted that we sorted various cell populations to ensure adequate representation; therefore, frequencies of cells observed in that experiment do not reflect their frequencies in the bone marrow. While our model of *M*. *avium*–induced trained immunity bears much in common with other models of trained immunity such as those using BCG vaccination or β-glucan (19, 20, 78), differences from model to model undoubtedly exist. While we focused on inflammatory pathways and effectors that are commonly induced in multiple murine models and human studies, more work is necessary to validate these findings in humans (19, 20, 78).

Overall, we provide one of the first studies to our knowledge to determine whether chromatin modifications induced in HSPCs are conserved through stages of myeloid differentiation. We identify 2 key gene targets that demonstrate epigenetic changes in common from the progenitor stage to fully differentiated Macs that may contribute directly to CTI.

## Methods

### Sex as a biological variable

Our previous work in *M*. *avium* infection found no significant differences in the mouse pathological responses to *M*. *avium*. Therefore, we used both sexes of mice for these experiments.

### Mice

For the trained immunity and sequencing experiments, both male and female C57BL/6 (CD45.2) and C57BL/6.SJL (CD45.1) mice were used, with equal number of males and females used per group per experiment. CD45.2 mice from ages 8–16 weeks were used for infection experiments, whereas CD45.1 from 6–9 weeks were used for initial competitive bone marrow transplants.

### THP-1 cell culture

THP-1 cells (ATCC TIB-2020) Cells were cultured in RPMI-1640 (Gibco, 11875093) supplemented with 10% FBS (Gibco, 10437-028) and 1% Penicillin (10,000 units/mL) and streptomycin (10,000 μg/mL) (Invitrogen, 15140122). THP-1 cells were passaged up to 20 times before stock was refreshed.

### Cell electroporation and CRISPR/Cas9

CRISPR/Cas9 electroporation was completed with the ThermoFisher neon transfection system. Cells were collected and washed 3 times with PBS and counted for viability. Scramble control was purchased from Synthego. GBP6 sgRNAs were generated through NCBI browser, while IRF1 sgRNAs were based on previous reported sgRNAs (85). Specific 5′–3′ sequences are listed in Supplemental Table 26. Generated single guide RNAs (sgRNAs) were used at 1 μg/μL, pooled together per group types and heated to 95°C for 2 minutes to open sgRNA complexes. Afterward, samples were moved onto ice for 2 minutes, followed by the addition Cas9 (Synthego 1 μg/μL) and incubated for 15 minutes at room temperature. Concurrently, 500,000 THP-1 cells were collected per sample, spun down, and resuspended in T buffer from the Neon transfection kit (ThermoFisher, MPK1025). Cells were then added to the Cas9/sgRNA mixture post-incubation, and electroporation was carried out following the Neon transfection system protocol. Electroporation conditions were 1600V with 10 ms spaces and 3 pulses.

### M. avium microbial infections

Mice were infected i.v. via retro-orbital injection with *M. avium* (2 × 10^6^ colony forming units [CFU]) as previously described. *M*. *avium* CFUs were quantified plating homogenates onto 7H10 Middlebrook agar plates (BD 262710) supplemented with OADC enrichment supplement (BD 212351).

### Bone marrow transplant and trained immunity challenge experiments

For competitive transplants, bone marrow from CD45.2 mice was isolated from harvested tibias, femurs, and pelvic bones and pooled per group. Cells are RBC lysed (BioLegend 420301) and cKit enriched via magnetic bead enrichment (Miltenyi 130-097-146). Cells were then sorted for lineage^–^ cells on a Sony Sorter SH800 with a 100 μM microchip. Live 20,000 CD45.2 Lin^–^ cKit enriched cells and 200,000 CD45.1 rescue marrow were transplanted into CD45.1 mice that had been lethally irradiated CD45.1 recipients (split dose 10 Gy from a Cesium Irradiator). CD45.2 donor cell engraftment is checked with flow cytometry on recipients’ peripheral blood every 4 weeks on a BD Fortessa.

For trained immunity challenge experiments, recipients were infected with 2.0 × 10^6^ CFU *M*. *avium*. After 4 weeks, spleens were harvested and plated as described below.

### M. avium bacterial plating

Spleens harvested from transplant recipients are weighed and checked for size. Half spleens were then weighed and crushed in media (HBSS, 2% FBS, 1% HEPES) and filtered into tubes at 10 mL resuspension. Volumes were measured by mass, with the assumption of density being 1 g/mL, and crushed spleen samples were serially diluted to 1 × 10^–3^ to 1 × 10^6^. In total 100 μL of serially diluted homogenate is plated in duplicate per dilution onto 7H10 agar plates supplemented with OADC enrichment (BD 262710, BD 212351). After plates are incubated for one week, plates are colony counted for bacterial colony forming unit (CFU) calculations.

### BMDM generation

Lin^–^, cKit-enriched hematopoietic cells were isolated from whole bone marrow via flow activated cell sorting (FACS). Cells were then plated and expanded in DMEM/F12/Glutamax (Gibco, 10565042) supplemented with 1% penicillin (10,000 units/mL) and streptomycin (10,000 μg/mL) (Invitrogen, 15140122), 10% FBS (Gibco, 10437-028), and 1 mL 1M HEPES (Gibco, 15630080). Cells were treated with both recombinant murine IL-3 (rm-IL3, Peprotech, 50ng/mL) and recombinant murine Mac CSF (rm-M-CSF, Peprotech, 50 ng/mL) to promote myeloid progenitor expansion and Mac expansion. Cells were maintained in a CO_2_ incubator at 37°C. Cells were transferred to a larger container to eventually be adherent in T75 flasks. Fresh media was added to the existing media per transfer to avoid overstressing cells in culture. Fresh cytokines were added at the same concentrations after each expansion, which happened every other day. After 1 week, cells were treated with only rm-M-CSF.

### Seahorse assay

Mature BMDMs were harvested and spun down at 1,200 rpm for 5 minutes at 4°C and resuspended in DMEM culture media for cell counting (Cellometer Auto 2000, Nexcelom, Lawrence, MA). BMDMs were then seeded in 8 well miniplates (Agilent, 103022-100) at a concentration of 7 × 10^4^ cells/well in 80 μL DMEM media and placed in a CO_2_ incubator at 37°C overnight to allow adherence. One day before the seahorse assay. Sensor cartridges (Agilent, 103022-100) were hydrated with 200 μL ultrapure water and placed in a non-CO_2_ incubator at 37°C overnight. On the day of the procedures, DMEM was removed from cell plates and washed with 200 μL basal media (Agilent Technologies, 103565-100) supplemented with 25 mM glucose (Agilent, 103578-100), 2 mM Na-Pyruvate (Agilent, 103577-100), and 2 mM L-glutamine (Agilent, 103579-100) for 3 times and replaced by supplemented basal media at a final volume of 175 μL. Sensor cartridge hydrated buffer was replaced by 200 μL Agilent calibration buffer. Various drugs from the Seahorse mitostress test kit (Agilent, 103010-100) were then dissolved in supplemented basal media, with final working concentrations being 1.5 μM Oligomycin, 0.75 μM FCCP, and 0.5 μM AA/Rotenone. Mitochondrial function was assessed on a Seahorse XFp; 8 well analyzer in real time following the injection of oligomycin, FCCP, and AA/Rotenone following the manufacturer’s instructions. Specific mitochondrial parameters were calculated according to the manufacturer’s protocol.

### Cytokine analysis

BMDMs were counted and seeded at 200,000 cells per well media (DMEM/F12/Glutamax, 10% FBS, HEPES) for 24 hours before the challenge. *M*. *avium* at a multiplicity of infection of 1 (MOI 1) was used to challenge the cells, and supernatant containing cytokines was collected 4 hours after challenge. The supernatant was sent to Eve Technologies (Calgary, AB, Canada) for targeted cytokine analysis with a Luminex 200 platform 10-Plex Discovery Assay via ELISA.

### CUT&RUN-Seq

CUT&RUN-Seq protocol was performed with based on published work from the lab and others (25, 86). Bone marrow from *M*. *avium–*infected or naive mice was isolated from tibias, femurs, and pelvic bones and pooled per group. After RBC lysis, cells were separated by cKit-enrichment and stained for specific cell surface markers. From cKit^+^ fractions, 30,000 HSCs (Lin^–^, cKit-enriched, CD150^+^, CD48^–^), 200,000 MPP3s (Lin^–^, cKit-enriched, CD150^–^, CD48^+^, Flk2^–^, CD34^+^), and 200,000 GMPs (Lin^–^, cKit-enriched, CD34^+^, CD16/32^+^) were sorted per sample. In total, 200,000 Macs (CD3^–^, B220^–^, NK1.1^–^, CD11b^+^, Ly6g^–^, Ly6c^+^) were sorted per sample from the cKit^–^ fractions. Samples were counted and washed with wash buffer — 50 mL H_2_O with 20 mM HEPES (Gibco, 15630080), 150 mM NaCl, 0.5 mM Spermidine, and 1 Roche Complete Protease inhibitor EDTA-free tablet (Sigma, 04693132001) — 3 times. Cells were then incubated with activated Concanavalin A beads (Bang Laboratories L2007231C) for 15 minutes at room temperature. Sample slurries were then washed and resuspended in wash buffer supplemented with 0.5% Digitonin (Dig-wash buffer). Dig-wash buffer was used for the remainder of the experimental method, with samples and buffers were kept on ice or at 4°C. Samples were incubated with specific antibodies overnight at 4°C on a rotator. Antibodies used were targeted for Rabbit anti-H3K27ac (Cell Signaling Technologies 8173, 1:50 concentration), Rabbit anti-H3K427me3 (Cell Signaling Technologies 9733, 1:50 concentration), and Rabbit anti-mouse IgG (Jackson ImmunoResearch 315-005-003, 1:50 concentration). After incubation, samples were washed 3 times and incubated with pAG-MNase (Epicypher SKU:15-016) on a rotator for 1 hour at 4°C. Samples were washed 3 times and then resuspended in 150 μL Dig-wash buffer. 2 μL of 100 mM CaCl2 was added to catalyze MNase activity and cutting, and reaction was kept at 4°C for 40 minutes. The reaction was quenched with 2X stopping buffer (5 mL total, H2O, NaCl 340 mM, EDTA 20 mM, EGTA, 4 mM, Digitonin 0.05%, RNase A 100 μg/mL, Glycogen 50 μg/mL, and Spike-in DNA 1 ng/mL; EpiCypher, 18-1401), and samples were heated to 37°C for 20 minutes for MNase/Histone/DNA complex release. Samples were placed on magnetic stands to separate the DNA supernatant from the bead slurry, and DNA purification was completed via phenol chloroform and ethanol precipitation extraction.

For CUT&RUN qPCR, DNA was detected by preamplifation using Invitrogen Platinum SuperFi II polymerase followed by qPCR using SybrGreen Supermix and GBP6-specific primers on a Quantstudio System. Data were normalized using a GAPDH control. Primers were: GBP6 forward primer (5′ TCGAGAGTTCCATCTTGCAGGC 3′), GBP6 reverse primer (5′ AGCAGCTCTTGCTCCTTCTCTG 3′), GAPDH forward primer (5′ CAGGAGAGTGTTTCCTCGTCC 3′), GAPDH reverse primer (5′ TTCCCATTCTCGGCCTTGAC 3′).

For CUT&RUN-Seq, libraries were generated with the services of AdmeraHealth. Quantified DNA by Qubit 2.0 HS DNA (ThermoFisher, Q32851) was assessed by Tapestation High on a D1000 High sensitivity DNA Assay (Agilent Technologies). Library preparation was completed using the KAPA HyperPrep kit (Roche) per the manufacturer’s instructions. Library quality was assessed with Qubit 2.0 (ThermoFisher), Tapestation (Agilent Technologies), and QuantStudio 5 System (Applied Biosystems). Illumina 8 nt dual indices were used. Libraries were pooled equimolarly based on quality and sequenced on an Illumina NovaSeq (Illumina) with a 150 paired-end (PE) read length.

Samples were prepared in biological duplicates, while one negative IgG control was generated per cell type and cell condition.

### scATAC-Seq library generation and sequencing

CD45.2 mice were infected with 2 × 10^6^ CFU *M*. *avium* for 4 weeks prior to bone marrow isolation. Isolated bone marrow from *M. avium*–infected and naive mice were collected, pooled by sample group, RBC-lysed, and enriched for cKit^+^ cells. Specific HSPCs, including LT-HSCs (Lin^–^, cKit^+^, CD48^–^, CD150^+^, Flk2^–^, CD34^–^), CD41^+^ HSCs (Lin^–^, cKit^+^, CD150^+^, CD41^+^), MPP3s (Lin^–^, cKit^+^, CD48^+^, CD150^–^, Flk2^–^, CD34^+^), and GMPs (Lin^–^, cKit^+^, CD41^–^, CD16/32^+^), and lineage cells, including Macs (B220^–^, CD3^–^, CD11c^–^,Gr1^+^, Mac-1^+^, Ly6g^–^, Ly6c^+^), neutrophils (B220^–^,CD3^–^, CD11c^–^, Gr1^+^, Ly6g^+^, Ly6c^+^), and B cells (B220^+^, CD3^–^, Gr1^–^, Mac-1^+^), were stained and sorted for with FACS on BD Aria IIs. Cells were counted and combined in equal ratios per group based on live/dead counts after sort at roughly 50,000 cells per group. Nuclei were isolated and loaded onto the Chromium controller system to generate 10X scATAC-Seq libraries. Library preparation and generation was handled by the Baylor Single Cell and Genetics core, and quality control of libraries was completed using a Bioanalyzer and Nanodrop system. Nuclei were isolated prior to analysis on the 10x genomics platform.

### Statistical analysis and bioinformatics

#### Statistics.

All statistical methods and handling of data was performed in GraphPad Prism v10.0. Mean with standard deviations for all data figures was used in figures. Individual data points per experiment were assessed for outliers using the ROUT method (Q = 5%). Collective data was then checked for Gaussian Distribution with the Shapiro-Wilk test. Two-tailed Student’s *t* test, ordinary 1-way ANOVA with Tukey’s multiple comparison, or 2-way ANOVA were used per experiment. Experiments were run at least twice unless stated, and the number of replicates and further statistical details are provided in the figure legends.

#### CUT&RUN-Seq.

FASTQ files were aligned to murine genome mm10 using the BWA aligner in Illumina Basespace, to which files are aligned with bwa and converted into aligned BAM files via samtools. BAM files were used for downstream analysis. MACS2 (v.2.2.7.1) (87) was used for peakcalling using the –broad –q 0.01 functions in addition to standard parameters, with test samples compared with background IgG controls. Bedgraph files produced by MACS2 were used for genomic track visualization using EaSeq (88) and confirmed with integrative genomic viewer (IGV).

Significant peaks were then identified and used for differential analysis using the Diffbind R package (v3.14) (89). Samples were filtered based gene blacklists and IgG greylists, and count matrixes were then generated and normalized by default Diffbind parameters (library size). Differential analysis was performed with cutoffs log_2_FC > 1 and FDR cutoff < 0.05. Chromosomal regions with significant peaks were then filtered based on fold changes that were positive and negative, to which cis-associated gene identification and gene ontogeny were performed GREAT (v4.04) (90) and rGREAT(v2.6) (91) Gene ontogeny were considered significant if the calculated BinomialFDR < 0.05.

Normalized count matrixes produced by Diffbind were extracted and used to produce clustering heatmap plots based on *z* scores using DESeq2 (v1.44.0) (92).

#### scATAC-Seq.

Quality control was performed prior to statistical analyses to remove low quality cells. Retained cells required between 500 and 30,000 peak region fragments, at least 25% reads in peaks, a ratio of genomic reads in blacklist regions less than 0.1, a ratio of mononucleosomal to nucleosome-free fragments less than 2.5, and a TSS enrichment score greater than 1.5. The scATAC-Seq datasets from *M*. *avium*–infected and naive mice were then integrated to maintain a consistent set of peaks across the 2 datasets. A common peak set was defined using the GRanges reduce function and quantified for each dataset using the FeatureMatrix Seurat function. The datasets were then embedded into a shared low-dimensional space using a reciprocal Latent Semantic Indexing projection.

Cell type labels were predicted by mapping the gene activity computed by the Signac R package to previously published scRNA-Seq data. The cell type annotation for the scRNA-Seq dataset used the Haemopedia Mouse RNA-Seq Atlas (v1.5) (93) and Immgen RNA-Seq profiles (v1.2) (94) as reference datasets to predict cell type labels using the MapQuery Seurat function. To refine the peaks analyzed in the dataset, we performed peak calling using MACS2 via the CallPeaks Signac function with cell type as a grouping variable. These new peaks were combined into a common peak set and quantified across all cells.

Differential accessibility (DA) analysis comparing chromatin openness in *M*. *avium*–infected mice relative to naive mice was performed for each cell type individually, as well as overall. Both positive and negative markers were found using the FindMarkers Seurat function utilizing the logistic regression framework with the total number of fragments as a latent variable. Peaks that were present in at least 5% of the cells in either population were included in the DA analysis. Motif enrichment analysis with background peaks matched for overall GC content was performed to identify DNA motifs overrepresented in peaks that were significantly more accessible (*P* < 0.005) in *M*. *avium*–infected mice and naive mice.

All analyses were performed in R (v4.4.1) using the Seurat (v5.1.0) (95) and Signac (v1.14.0) (96) R packages.

### Study approval

The handling and maintenance of mice complied with the protocols and guidelines approved by the IACUC at Baylor College of Medicine.

### Data availability

All biological materials, reagents, sgRNAs, kits, and bioinformatic tools utilized for these experiments are listed in Supplemental Table 26. Epigenetic datasets are available in GEO, and accession nos. are provided in Supplemental Table 26. Values for all data points in graphs are reported in the Supporting Data Values file.

## Author contributions

BTT and KYK designed the study, conducted the experiments, and wrote the paper. PNL performed bioinformatic analyses, provided the relevant interpretations, and wrote the paper. RC and SK performed the BMDM functional experiments and analysis. DTL contributed to the CUT&RUN-Seq experiments. BNK designed the scATAC-Seq experiments. AT designed optimized flow cytometry panels for FACS and performed data analysis. LM and AC provided assistance with Seahorse Assays. Coauthor order was agreed by the 2 coauthors prior to manuscript writing.

## Funding support

This work is the result of NIH funding, in whole or in part, and is subject to the NIH Public Access Policy. Through acceptance of this federal funding, the NIH has been given a right to make the work publicly available in PubMed Central.

- Baylor College of Medicine Single Cell Genomics
- NIH 1S10OD018033
- Cytometry and Cell Sorting Core at Baylor College of Medicine
- CPRIT Core Facility Support Award (CPRIT-RP180672)
- NIH (P30 CA125123 and S10 RR024574)
- Cancer Prevention and Research Institute of Texas (CPRIT-RR140038; to AC)
- American Society of Hematology (Bridge grant 2023, round 20)
- Cell and Gene Therapy Training Grant T32HL2332 (BTT)
- F31HL164287 (BTT)
- Hematology Training Grant 5T32DK06445 (AT)
- F30HL172468 (AT)
- P01CA265748 (KYK)
- R35 HL155672 (KYK).

## Supplementary Material

Supplemental data

Supplemental tables 1-7

Supplemental tables 18-21

Supplemental tables 22-25

Supplemental table 26

Supplemental tables 8-17

Supporting data values

## Figures and Tables

**Table 1:**
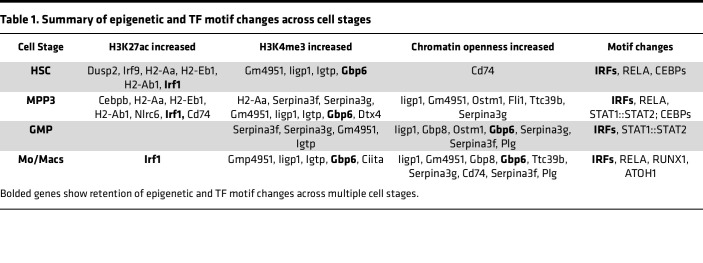
Summary of epigenetic and TF motif changes across cell stages

## Version 1 12/09/2025

In-Press Preview

## Version 2 01/23/2026

Electronic publication
