# Supplementary material for: Rare epigenetic alterations are conserved across hematopoietic differentiation stages after mycobacterial infection
Source: JCI Insight. 2025 Dec 9;11(2):e193686. doi: 10.1172/jci.insight.193686 (PMC12892905; doi:10.1172/jci.insight.193686)
Supplement: Supplemental data [file jciinsight-11-193686-s247.pdf]

Supplemental Figures

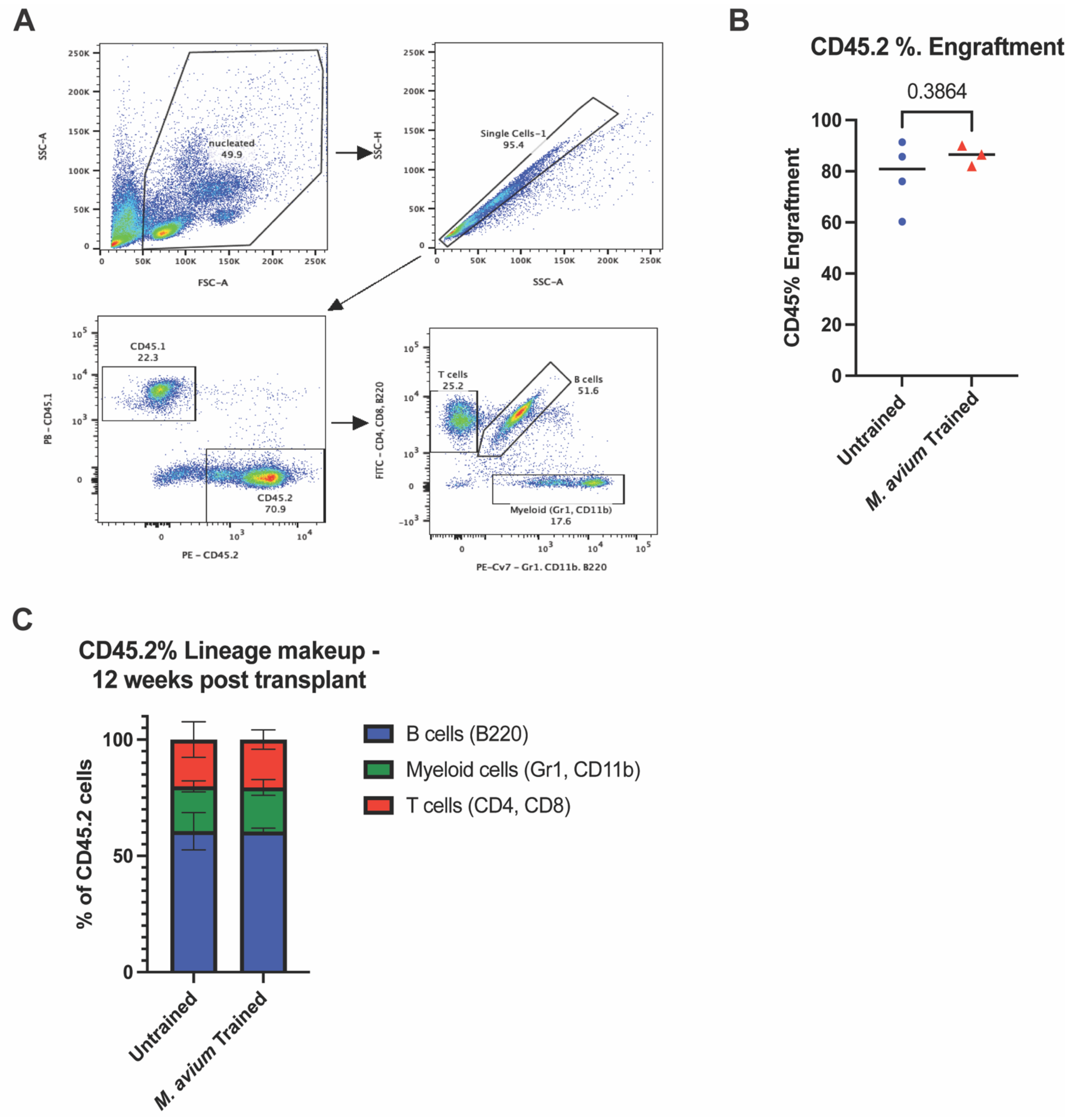

Supplemental Figure 1: Analysis of transplant engraftment of trained immunity transplant experiments.

(A) Peripheral blood engraftment flow cytometry gating strategy to identify donor distinct cells and trilineage production. **(B)** Percentage of CD45.2 bone marrow compared to total CD45.2 and CD45.1

cells. Representative engraftment of two independent experiments. **(C)** Lineage breakdown of CD45.2 positive cells within untrained and *M. avium* trained groups. Figure representative of two independent experiments. Unpaired Student's t test was used for **(B)**, showing no significant differences in CD45.2 engraftment.

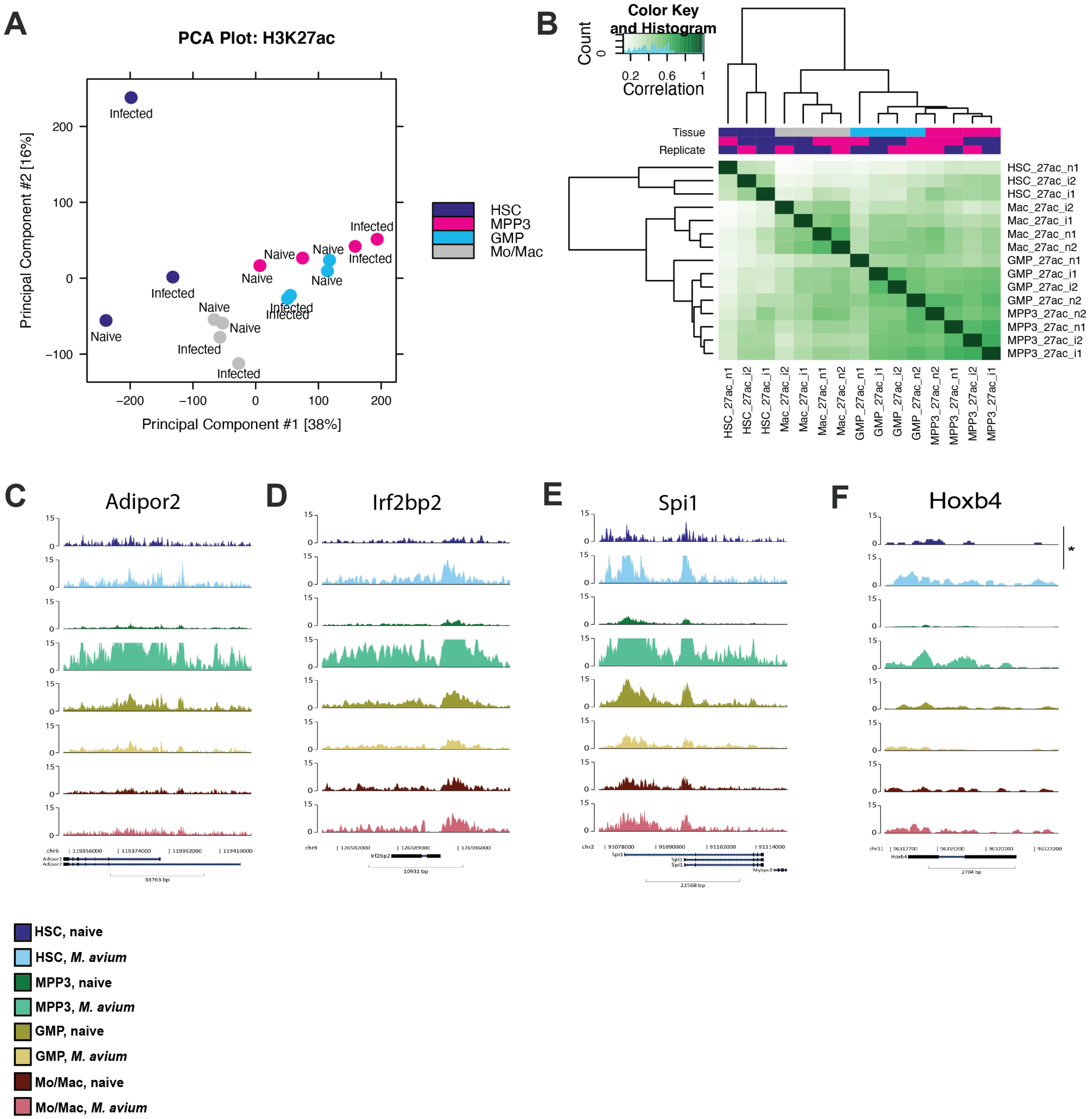
A
PCA Plot: H3K27ac
Principal Component #2 [16%]
Principal Component #1 [38%]
200
100
0
−100
−200
−100
0
100
200
Infected
Naive
HSC
MPP3
GMP
Mo/Mac
B
Color Key and Histogram
Count
0
0.2
0.6
1
Correlation
Tissue
Replicate
HSC_27ac_n1
HSC_27ac_i2
HSC_27ac_i1
Mac_27ac_i2
Mac_27ac_i1
Mac_27ac_n1
Mac_27ac_n2
GMP_27ac_n1
GMP_27ac_i1
GMP_27ac_i2
GMP_27ac_n2
MPP3_27ac_n2
MPP3_27ac_n1
MPP3_27ac_i2
MPP3_27ac_i1
C
Adipor2
D
Irf2bp2
E
Spi1
F
Hoxb4
15
0
*
33763 bp
10931 bp
22568 bp
2704 bp
HSC, naive
HSC, M. avium
MPP3, naive
MPP3, M. avium
GMP, naive
GMP, M. avium
Mo/Mac, naive
Mo/Mac, M. avium

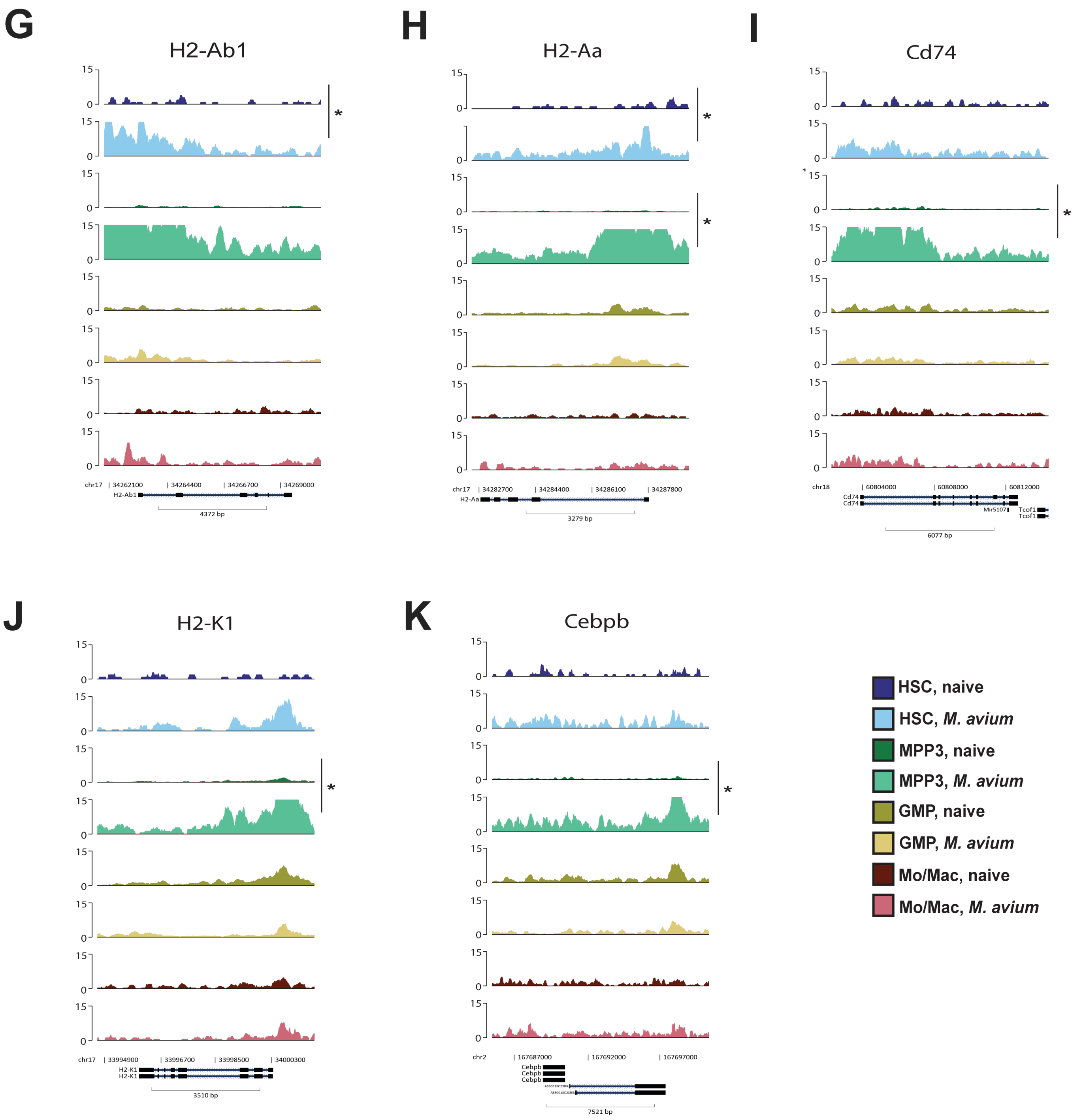

Supplemental Figure 2: Analysis of CUT&RUN-sequenced H3K27ac libraries and genomic coverage from *M. avium* infected and naive samples.

(A) PCA Plot of H3K27ac libraries in both *M. avium* infected and naïve groups **(B)** Pearson correlation plot of H3K27ac sample libraries **(C-D)** Coverage plots of *Adipor2* **(C)**, *Irf2bp2* **(D)**, *Spi1* **(E)**, and *Hoxb4* **(F)**. Comparisons marked with an asterisk were found to be significant at least at FDR

< 0.05.**(G-K)** Coverage plots of *H2-Ab1* **(G)**, *H2-Aa* **(H)**, *Cd74* **(I)**, *H2-K1* **(J)**, and *Cebpb* **(K)**.

Comparisons marked with an asterisk were considered significant at least at an FDR < 0.05.

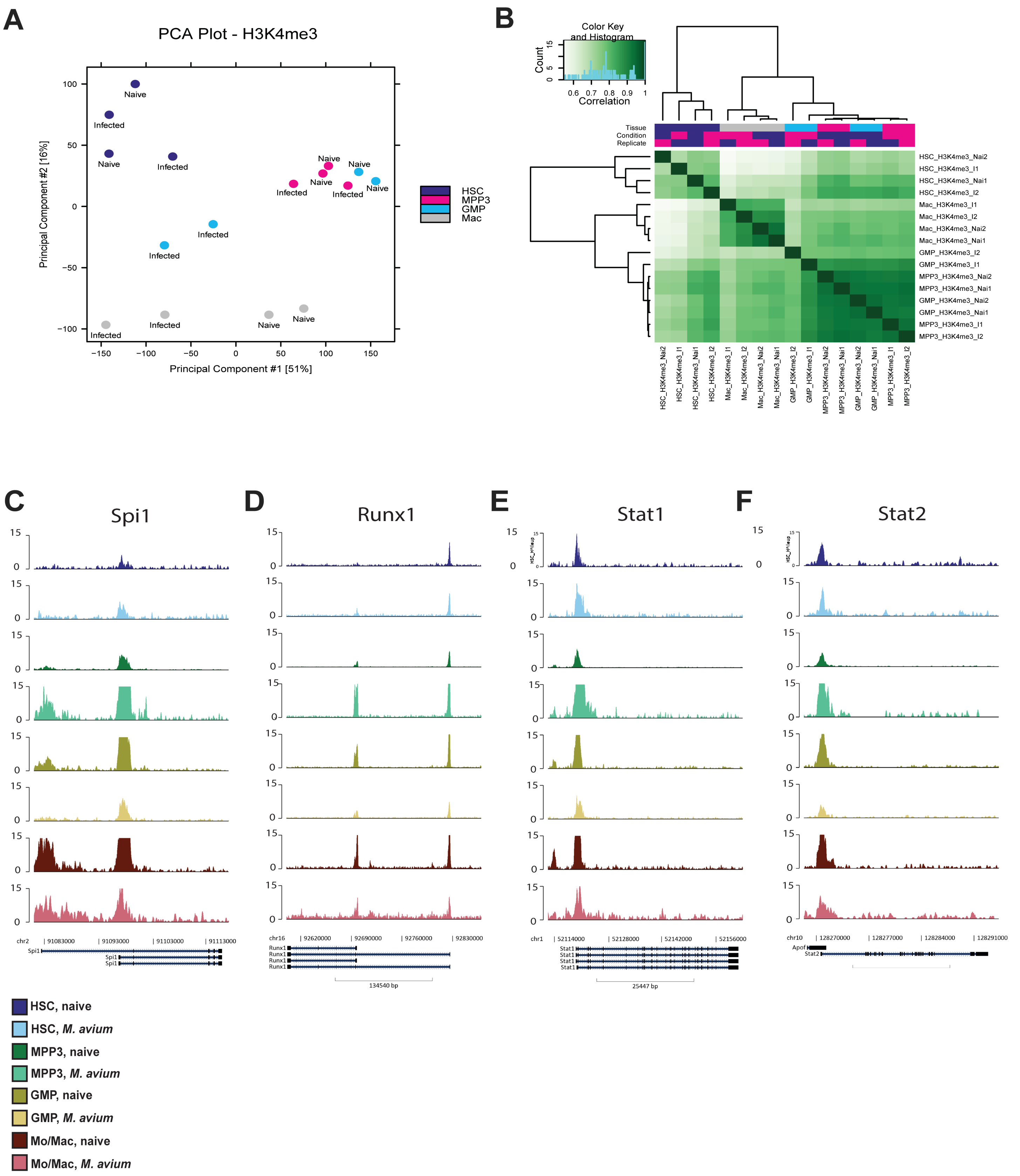
A
PCA Plot - H3K4me3
Principal Component #1 [51%]
Principal Component #2 [16%]
Naive
Infected
HSC
MPP3
GMP
Mac
B
Color Key and Histogram
Count
Correlation
Tissue
Condition
Replicate
HSC_H3K4me3_Nai2
HSC_H3K4me3_I1
HSC_H3K4me3_Nai1
HSC_H3K4me3_I2
Mac_H3K4me3_I1
Mac_H3K4me3_I2
Mac_H3K4me3_Nai2
Mac_H3K4me3_Nai1
GMP_H3K4me3_I2
GMP_H3K4me3_I1
MPP3_H3K4me3_Nai2
MPP3_H3K4me3_Nai1
GMP_H3K4me3_Nai2
GMP_H3K4me3_Nai1
MPP3_H3K4me3_I1
MPP3_H3K4me3_I2
C
Spi1
D
Runx1
E
Stat1
F
Stat2
chr2
chr16
chr1
chr10
134540 bp
25447 bp
Apof
HSC, naive
HSC, M. avium
MPP3, naive
MPP3, M. avium
GMP, naive
GMP, M. avium
Mo/Mac, naive
Mo/Mac, M. avium

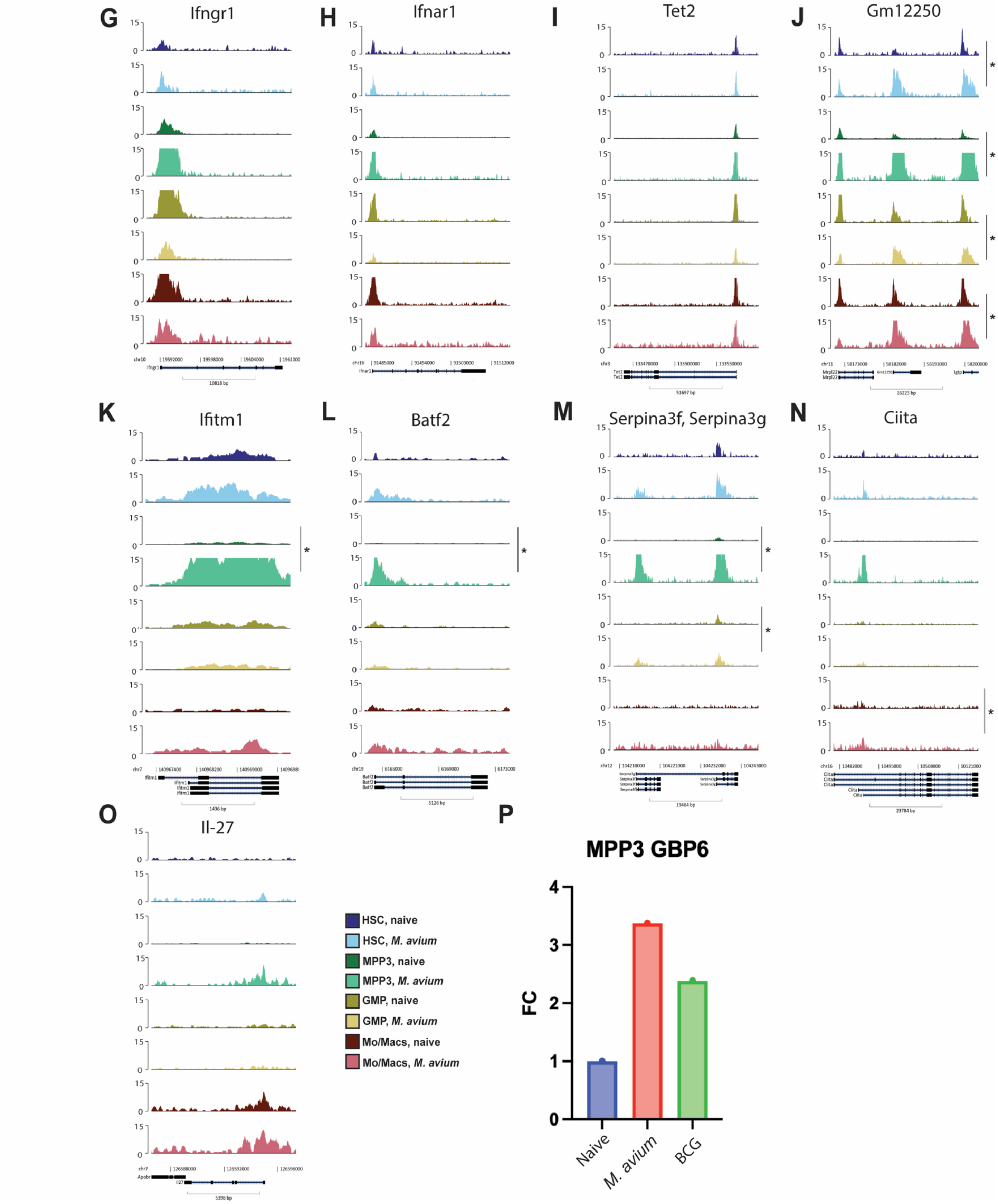

Supplemental Figure 3: Analysis of CUT&RUN-sequenced H3K4me3 libraries and genomic coverage from *M. avium* infected and naive samples.

(A) PCA Plot of H3K4me3 libraries across cell types across naïve and *M. avium* infected conditions **(B)** Pearson correlation plot of H3K4me3 libraries across cell types in naïve and *M. avium* infected conditions **(C-F)** Coverage plots of *Spi1* **(C)**, *Runx1* **(D)**, *Stat1* **(E)**, and *Stat2* **(F)**. Comparisons marked with an asterisk were considered significant at least at an FDR < 0.05 **(G-O)** Coverage plots of *Ifngr1* **(G)**, *Ifnar1* **(H)**, *Tet2* **(I)**, *Gm12250* **(J)**, *Ifitim1* **(K)**, *Batf2* **(L)**, *Serpina3f*, *Serpina3g* **(M)**, *Ciita* **(N)**, and *Il-27* **(O)** Comparisons marked with an asterisk were considered significant with at least an FDR < 0.05. **(P)** Relative H3K4me3 marks at GBP6 in MPP3s after 2-week *M. avium* or BCG exposure.

A

Sorted cell populations	Cellular fraction	Phenotype
LT-HSCs	c-kit +	Lin$^-$ c-kit$^+$ CD48$^-$ CD150$^+$ Flk2$^-$ CD34$^-$
MPP3	c-kit +	Lin$^-$ c-kit$^+$ CD48+ CD150- Flk2$^-$ CD34$^+$
GMP	c-kit +	Lin$^-$ c-kit$^+$ CD41$^-$ CD16/CD32+
CD41+ HSCs	c-kit +	Lin$^-$ c-kit$^+$ CD48$^-$ CD150$^+$ CD41$^+$ (Gekas and Graf Blood 2013)
Inflammatory Macrophages	c-kit -	B220$^-$ Gr1$^+$ Mac-1$^+$ CD3$^-$ CD11c$^-$ Ly6g$^-$ Ly6c$^+$
Neutrophils	c-kit -	B220$^-$ Gr1$^+$ Mac-1$^+$ CD3$^-$ CD11c$^-$ Ly6g$^+$
B cells	c-kit -	B220$^+$ Gr1$^-$ Mac-1$^-$ CD3$^-$

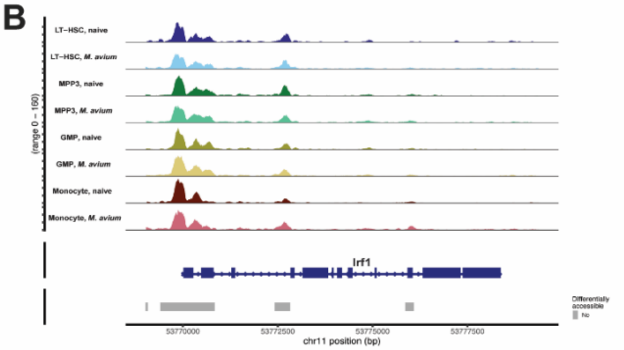

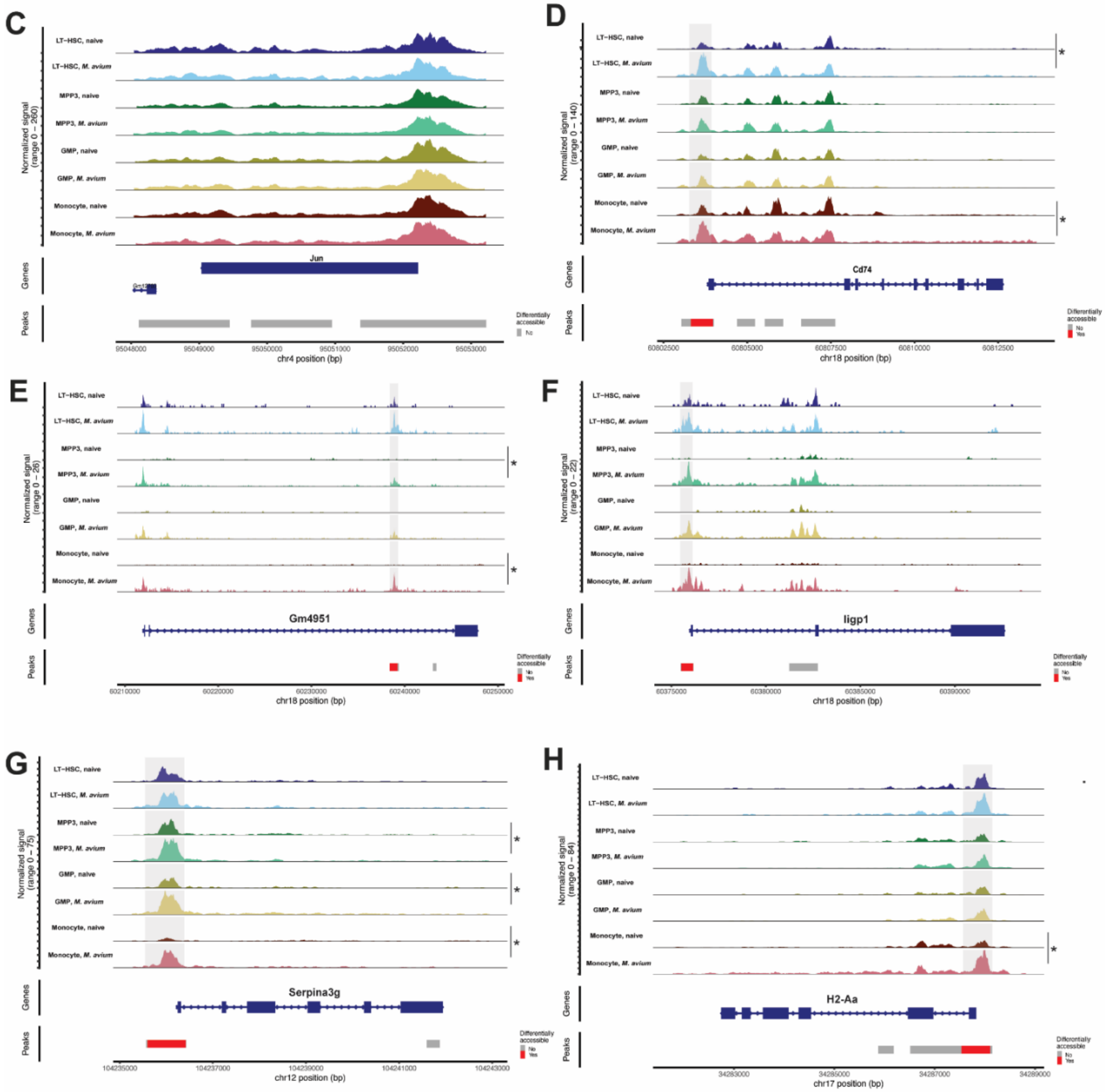

Supplemental Fig.4: Genomic coverage and peakcalling of chromatin accessibility across *M. avium* infected and naive cell populations.

(A) Cell sorting table indicating cell surface markers used to specially sort cell populations via FACS

(B-G) Genomic coverage plots of *Irf1* **(B)**, *Jun* **(C)**, *Cd74* **(D)**, *Gm4951* **(E)**, Iigp2 **(F)**, *Serpina3g* **(G)**, *H2-Aa* **(H).**

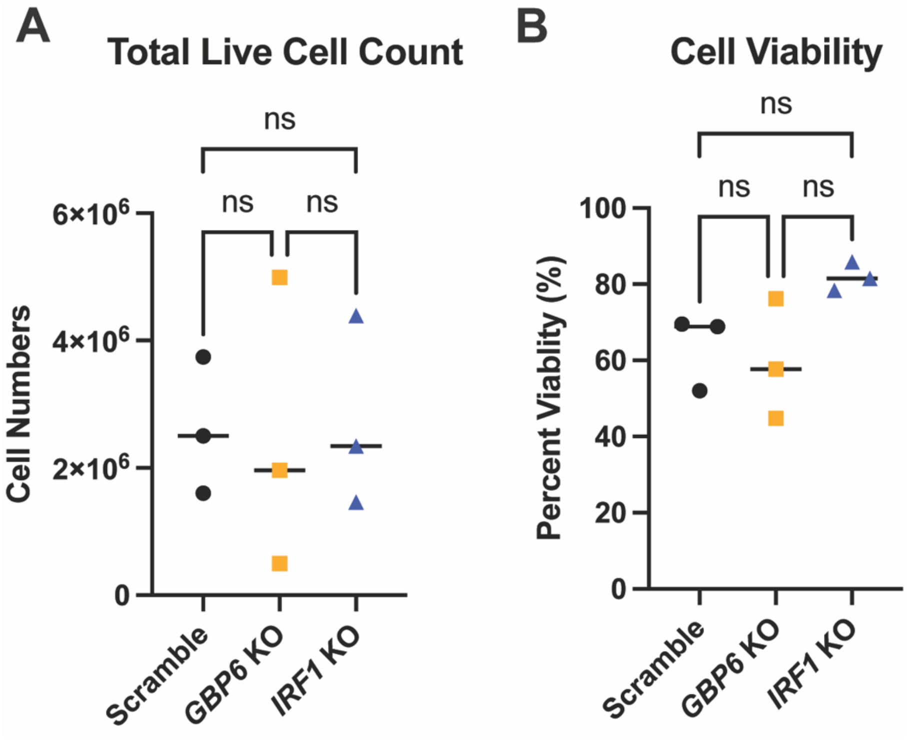

Supplemental Figure 5: PMA treatment of IRF1 KO and GBP6 KO THP-1 cells induces varying differentiation responses.

(A) Total live cell counts per group. Each dot represents the cell number from an independent experiment. n= 3 **(B)** Percent live cells based on AOPI staining of PMA-treated THP1 cells after three days stimulation. Each dot represents the percent cell viability from an independent experiment. n = 3 ns; not significant by one-way ANOVA.
